# Enhancing experimental design through Bayes factor design analysis: insights from multi-armed bandit tasks

**DOI:** 10.12688/wellcomeopenres.22288.1

**Published:** 2024-08-01

**Authors:** Sarah Schreiber, Danielle Hewitt, Ben Seymour, Wako Yoshida

**Affiliations:** 1 Institute of Biomedical Engineering, University of Oxford, Oxford, England, OX37DQ, UK; 2 Wellcome Centre for Integrative Neuroimaging, University of Oxford, Oxford, England, OX39DU, UK; 3 Department of Neural Computation for Decision-making, Advanced Telecommunications Research Institute International, Kyoto, Japan

**Keywords:** design calculation, Bayes factor, design analysis, sample size, statistical evidence, exploration, decision-making

## Abstract

Bayesian statistics are popular in human cognitive neuroscience research because they can incorporate prior knowledge. Although well established for retrospective analysis, the application of Bayesian methods to prospective analysis is less well developed, especially when used in combination with computational model-based analysis of behavioural data. It is therefore important to establish effective methods for testing and optimising experimental designs for these purposes. One potential framework for a prospective approach is Bayes factor design analysis (BFDA), which can be used alongside latent variable modelling to evaluate and visualise the distribution of Bayes factors for a given experimental design.

This paper provides a tutorial-style analysis combining BFDA with latent variable modelling to evaluate exploration-exploitation trade-offs in the binary multi-armed bandit task (MAB). This is a particularly tricky example of human decision-making with which to investigate the feasibility of differentiating latent variables between groups as a function of different design parameters. We examined how sample size, number of games per participant and effect size affect the strength of evidence supporting a difference in means between two groups. To further assess how these parameters affect experimental results, metrics of error were evaluated.

Using simulations, we demonstrated how BFDA can be combined with latent variable modelling to evaluate and optimise parameter estimation of exploration in the MAB task, allowing effective inference of the mean degree of random exploration in a population, as well as between groups. However, BFDA indicated that, even with large samples and effect sizes, there may be some circumstances where there is a high likelihood of errors and a low probability of detecting evidence in favour of a difference when comparing random exploration between two groups performing the bandit task. In summary, we show how BFDA can prospectively inform design and power of human behavioural tasks.

## Plain Language Summary

Optimising the design of experiments before collecting data is an important part of cost-efficient and responsible research. However, this is challenging as experimental designs and analysis methods get complicated, as there are many parameters and factors that need to be balanced. These include the sample size and the number of trials each participant completes, but also parameters that are more specific to the experiment. In this work, we show how the use of an approach called ‘Bayesian Factor Design Analysis’ can optimize the experimental design of a human decision-making task, whereby participants choose between two options with varying outcomes. The objective of this task is to compare information-seeking (‘exploratory’) behaviour between two groups, which is an important problem in cognitive sciences - proposed, for example, as a key mechanism underlying the development of chronic pain. However, before starting clinical studies, we should first identify how easily we can detect differences between patients and healthy controls, so we know how much data we need to collect. We show that this approach effectively allows estimating the average level of information-seeking behaviour in a group, as well as between groups, according to various parameters. The analysis can identify situations with a high probability of error and a low probability of successfully detecting a difference between two groups.

## Introduction

Designing an effective experiment can be challenging due to the number of parameters that need to be considered. One such parameter is the sample size, which is commonly determined by conducting power calculations. This process is an example of a prospective design analysis aimed at optimizing study outcomes. Usually, this is followed by a retrospective evaluation of the design after data collection to test the statistical significance of a result. To improve a study's effectiveness, it is recommended to conduct a thorough prospective design analysis rather than relying purely on a retrospective approach ( Gelman & Carlin, 2014). This can help optimize the use of available resources, which has become increasingly important considering recent concerns about 'research waste' ( Ioannidis *et al.*, 2014; Macleod *et al.*, 2014; Storz-Pfennig, 2017).

Traditionally, sample size determination methods rely on frequentist statistics, but there has been an ongoing critique among statisticians and methodologists regarding these approaches. Among the main concerns are the common misinterpretation of *p*-values and significance testing ( Benjamin *et al.*, 2018; Gigerenzer, 2004; Morrison & Henkel, 2017; Wagenmakers, 2007; Wagenmakers *et al.*, 2018). Concurrently, Bayesian methods are gaining recognition for their advantages, as they allow the incorporation of prior knowledge into statistical processes ( Jeffreys, 1935; Jeffreys, 1961; Wagenmakers, 2007). Other benefits of this approach include the potential to individually measure the evidence for both the null and alternative hypotheses arising from the data. These considerations have led to arguments favouring a more balanced approach between frequentist methods and Bayesian frameworks in statistical methodology. Thus, as Bayesian statistical methods gain popularity, it is crucial to have the necessary tools to perform a comprehensive Bayesian design analysis.

Schönbrodt and Wagenmakers proposed Bayes factor design analysis (BFDA) as a method for design analysis ( Schönbrodt & Wagenmakers, 2018). This framework is based on the Bayes factor, a continuous measurement weighing the evidence for one hypothesis over another ( Morey & Rouder, 2011). The Bayes factor can be compared to decision thresholds that indicate different strengths of evidence for the null hypothesis as well as the alternative hypothesis respectively ( Jeffreys, 1961). BFDA evaluates the distribution of the Bayes factors for a given experimental design, providing a powerful alternative to frequentist a-priori power analyses.

BFDA assumes a population with predetermined attributes and conducts simulations on repeated sampling from this population ( Schönbrodt & Wagenmakers, 2018). For each sample, the comparative evidence between the null hypothesis and the alternative hypothesis is measured by calculating the Bayes factor. This method can be used to evaluate different design approaches in Bayesian statistics. In this work, we focus on what Schönbrodt and Wagenmakers call a fixed- *n* design, where each analysed sample is of a fixed sample size.

Previous literature on BFDA presents examples on how this approach can be used to calculate the probability of errors and how the sample size affects the probability of obtaining a Bayes factor of a certain value ( Schönbrodt & Wagenmakers, 2018; Stefan *et al.*, 2019; Stefan *et al.*, 2024). These examples are based on variables that are directly measurable. However, in psychology and neuroscience, we often deal with latent variables that first need to be inferred from the data collected. This is getting more common given the increasing popularity of fitting computational models to behavioural data.

Here, we consider an example problem of differentiating different levels of exploratory choices based on learned values, using the application of reinforcement learning models. This is a problem that directly relates to computational models of neurological and psychiatric disease, including chronic pain and depression ( Krypotos *et al.*, 2022a). For instance, in the classic ‘Fear Avoidance’ model of chronic pain, individuals with acute or subacute musculoskeletal pain are proposed to excessively avoid engaging in physical activity as they approach the recovery period, because of a failure to adequately explore movement actions that might no longer be as painful as expected ( Vlaeyen *et al.*, 2016). This leads to a cycle of inactivity and physical deconditioning, which itself ultimately worsens pain. However, the hypothesis as to whether this genuinely relates to impaired exploratory behaviour has not been tested, as it ideally requires a model-based analysis of exploratory behaviour, considering various confounding factors.

To do this, we first analysed the accuracy of Bayesian parameter estimations of latent variables, within a population, as well as between two groups, using simulated behavioural data in a multi-armed bandit task ( Krypotos *et al.*, 2024). In a second step, we combined BFDA with simulations of behavioural data to explore the relationship between sample size and the strength of evidence. This included examining the probability of substantial evidence for an incorrect hypothesis and the probability of insufficient evidence for the correct hypothesis. We also considered how other factors and considerations of realistic experimental design affect these properties. 

## Methods

### Bayes factor design analysis

***Bayesian statistics.*** Bayesian statistics is a method of combining evidence from observed data with prior information and beliefs ( Bayes, 1991; Kruschke, 2015a; Kruschke *et al.*, 2012). This means we are updating our beliefs based on new information in a probabilistic manner, considering the uncertainty of our prior beliefs as well as the collected data. Bayes' theorem describes this idea mathematically, as


$$P(\theta \text{|}Data)=\frac{P(Data\text{|}\theta )P(\theta )}{P(Data)}.\;\text{\#}(1)$$

Here *θ* represents the parameter being estimated, which generates the collected dataset. The resulting posterior distribution *P*( *θ*| *Data*) approximates the true probability distribution of *θ* on the basis of our prior beliefs and the available data. The prior distribution *P*( *θ*) represents our knowledge about the distribution of *θ* ahead of data acquisition. The chosen prior can heavily influence the outcome of an analysis and should therefore be chosen carefully, as it will bias the estimation. The likelihood *P*( *Data*| *θ*) quantifies the probability of measuring the observed dataset for various values of the unknown parameter we are aiming to estimate.

To calculate the posterior according to ( 1) we need the marginal density *P*( *Data*), which is calculated by


$$P(Data)=\int_{\theta }P(Data\text{|}\theta )P(\theta ).\;\text{\#}(2)$$

This results in the posterior


$$P(\theta \text{|}Data)=\frac{P(Data\text{|}\theta )P(\theta )}{\int_{\theta }P(Data\text{|}\theta )P(\theta )}.\;\text{\#}(3)$$

The marginal density *P*( *Data*) is therefore a simple normalizing constant and the posterior depends only on the likelihood *P*( *Data*| *θ*) and the prior *P*( *θ*).

The likelihood *P*( *Data*| *θ*) can be calculated based on the experimental design. Provided that the collected dataset *Y* consists of *n* independent measurements, *Y* = [ *y*_1_, *y*_2_, ..., *y _n_*], the likelihood of measuring this dataset is the product of the likelihoods of measuring each independent data point within the dataset


$$P(Y\text{|}\theta )=\prod_{i=1}^{n}P(y_{i}\text{|}\theta ).\;\text{\#}(4)$$

The likelihood function for each independent data point is specific to the experimental setup, and the chosen outcome measure.

From the posterior, a point estimate of *θ* can be calculated using


$$\hat{\theta }=\int P(\theta \text{|}Data)\theta \;d\theta .\;\text{\#}(5)$$

***Bayes factor.*** Once the likelihoods have been calculated for each group, we can compare *θ* between two groups, for example between patients and healthy controls. A Bayesian approach can be used to estimate this difference. Using the individual likelihoods *P*( *Y*_1_| *θ*_1_) and *P*( *Y*_2_| *θ*_2_) calculated as previously described, we can compute the probability distribution of the difference in *θ* between groups, *Δθ* = *θ*_1_ – *θ*_2_. Considering two independent continuous random variables *X* and *Y*, the probability density function of the difference *Z* = *X* – *Y* can be calculated by convolving the respecting probability density functions *f _Z_* ( *z*) = ∫ *f _X_* ( *x*) *f _Y_* ( *x–z*) *dx*. In our case we can calculate the convolution of the two likelihoods to get the probability density function of the difference


$$P(Y\text{|}\Delta \theta )=\int P(Y_{1}\text{|}\theta_{1})P(Y_{2}\text{|}\theta_{1}-\text{Δ}\theta )d\theta_{1}.\;\text{\#}(6)$$

This probability density function can then be used to obtain a point estimator for the difference in *θ* between the two groups. If the objective is to determine whether a difference exists, regardless of the exact value, we can calculate the Bayes factor


$$BF_{10}=\frac{P(Y\text{|}H_{1})}{P(Y\text{|}H_{0})},\;\text{\#}(7)$$

which is a mathematical description of Bayesian hypothesis testing ( Jeffreys, 1935; Jeffreys, 1961; Johnson *et al.*, 2023; Kruschke, 2015b). The Bayes factor weighs the evidence for one hypothesis against another. In our case, we are comparing the hypothesis that there is a difference in *θ* between two groups, *H*_1_: *Δθ* ≠ 0, against the null hypothesis, *H*_0_: *Δθ* = 0. Thus, our Bayes factor is calculated as


$$BF_{10}=\frac{\int P(Y\text{|}\Delta \theta )P(\Delta \theta )d\Delta \theta }{P(Y\text{|}\Delta \theta =0)}.\;\text{\#}(8)$$

**Figure 1.**
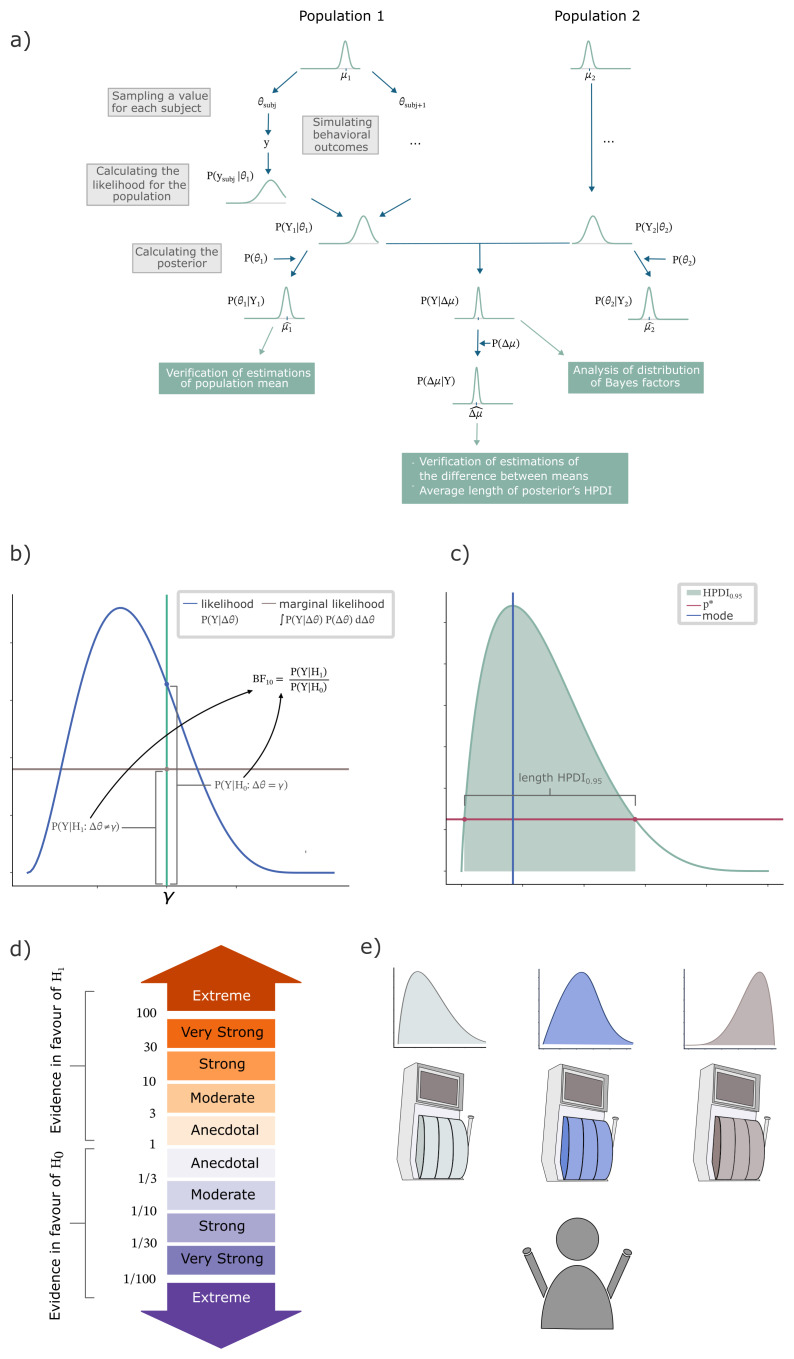
Summary of methods. BFDA was combined with latent variable modelling for an example of a MAB task. ( **a**) Repeated samples were taken from two populations and used to simulate behavioural outcomes of individual subjects. Based on these simulations the difference in means between the two groups was analysed by examining the distribution of Bayes factors, as well as probabilities of errors in the estimations of the difference. ( **b**) The Bayes factor was used as a measure of evidence in favour of a difference in mean. ( **c**) Example of a 95% HPDI. ( **d**) Decision thresholds for the Bayes factor indicating strengths of evidence for either hypothesis ( Jeffreys, 1935; Jeffreys, 1961; Kass & Raftery, 1995). ( **e**) A MAB task was used to illustrate how BFDA optimizes experimental design. The agent is presented with multiple options with different outcome probabilities. After each choice, the agent observes the immediate outcome before making the next choice.

A visual representation of the Bayes factor is shown in Figure 1b. An advantage of the Bayes factor is that it is a continuous measurement on the evidence for one hypothesis over another. A higher factor corresponds to stronger evidence in favour of our alternative hypothesis, just as a smaller factor of less than one corresponds to stronger evidence in favour of the null hypothesis. A widely used scale for categorising the Bayes factor is based on work by Jeffreys ( Jeffreys, 1935; Jeffreys, 1961; Kass & Raftery, 1995) and is shown in Figure 1d.

***Evaluation measurements.*** To give some examples of the benefits of using modelling in the design stages of an experiment, behavioural data from a multi-armed bandit task was simulated. An analysis was conducted to investigate the impact of sample size, number of games per participant, and difference in population mean on the computed Bayes factors. When considering the Bayes factor, it is important to note that it can have a significant variance ( Pfister, 2021). This means that the same experimental design and analysis will produce Bayes factors of different strengths of evidence when replicated. This is the fundamental principle of BFDA. It involves repeated sampling and analysis of a simulated population followed by an analysis of the distribution of Bayes factors across these samples ( Schönbrodt & Wagenmakers, 2018). To compare distributions for different experimental conditions, we can examine the frequencies of Bayes factors exceeding common thresholds, as outlined in Figure 1d. We can achieve this by running a set of simulations and calculating the average number of simulations with a Bayes factor indicating each of these strengths of evidence ( Figure 1a).

A further analysis evaluates the accuracy of the estimation of the difference between two groups by assessing the estimation error and the average length of the highest probability density interval (HPDI) to aid in determining an optimal sample size. HDPI is a method of obtaining a credible interval for skewed probability distributions. Instead of creating a symmetrical confidence interval around the mode of a probability density function, it chooses an interval that includes the highest posterior densities


$$C_{1-\alpha }=\{\theta :p(\theta \text{|}Y)>p^{*}\}.\;\text{\#}(9)$$

An example of a HPDI is shown in Figure 1c. The HPDI includes all values of *θ* that have a corresponding likelihood of *P*( *θ*| *Y*) > p*. Here, p* is chosen, so that


$$\int_{C_{1-\alpha }}P(\theta \text{|}Y,n)d\theta =1-\alpha .\;\text{\#}(10)$$

The average length of the posterior’s *HPDI* can be calculated in a two-step approach ( Joseph & Bélisle, 1997). Let *l'* be the length of the *HPD*_1–α_ interval for a given dataset *Y _i_*. The average length l* can be calculated from *l'* by multiplying it by the probability of *Y _i_* being the outcome for this measurement and integrating over all possible outcomes *γ* = [ *Y*_1_, *Y*_2_,...]


$$l^{*}=\int_{ϒ}l^{\prime }(Y,n)P(Y,n)dY.\;\text{\#}(11)$$

*P*( *Y,n*) is the predictive posterior and can be computed as


$$P(Y,n)=\int_{\theta }P(Y\text{|}\theta ,n)P(\theta )d\theta .\;\text{\#}(12)$$

Both the length of the HPDI and the predictive posterior are functions of the sample size of each group *n*. A higher *n* leads to a smaller HPDI, which indicates a higher certainty about the value of the estimated parameter.

In addition to serving as a tool for monitoring the quality of the estimation algorithm, the average length can also be used as a method for determining the sample size by predefining a maximum length. This is the basis of the average length criterion (ALC) ( Joseph & Bélisle, 1997). The sample size is determined as the smallest *n*, for which the average length of the HPDI is smaller than a set maximum length *l _max_*


$$\int_{ϒ}l^{\prime }(Y,n)P(Y,n)dY\leq l_{max}.\;\text{\#}(13)$$

Other common methods for sample size determination include the average coverage criterion, and the worst outcome criterion ( Cao *et al.*, 2009).

### The exploration-exploitation dilemma

***Multi-armed bandit task.*** To validate the pipelines for the described approach and to give an example, a binary two-armed bandit task was simulated. The MAB is a common task to research the exploration-exploitation dilemma in behavioural science ( Danwitz *et al.*, 2022; Daw *et al.*, 2006; Gershman, 2019), in which participants are presented with a series of options, each of which has multiple possible outcomes with different probabilities ( Figure 1e). After choosing one option, also referred to as an ‘arm’, participants observe an immediate outcome and are free to make the next choice. They repeat this for *x* trials before the game ends and the reward probabilities change for the next round. To maximize the number of rewarding outcomes throughout a game, the participant must find the right balance between exploring the possible options to try to determine the outcome probability for each option and using the information they have gathered so far to choose the option they think has the highest probability of a positive outcome. The binary version of the MAB task is often used in pain research, where the two possible outcomes are a painful stimulus and the absence of a painful stimulus ( Krypotos *et al.*, 2022a; Krypotos *et al.*, 2022b).

***Explore-exploit trade-off in the Horizon task.*** In the specific task that was implemented, the agent has information on both options available to them prior to their first choice. This is achieved by presenting the agent with four actions and their immediate results, after which they are free to make their own choices. This approach facilitates the differentiation between exploration and exploitation, as the agent possesses information to base their exploitation on ( Wilson *et al.*, 2014). The task was implemented for a horizon of 10, allowing the agent 6 free choices after observing the initial 4 outcomes. Each game consisted of one round of 4 observed trials and 6 free choice trials, after which the probability of an aversive outcome for each arm changed. The outcome probabilities were combinations of the probabilities 0.1, 0.3, and 0.9.

Calculating the likelihood of a certain outcome in this case is somewhat complex, as we must model human behaviour. If we want to infer a participant’s tendency towards exploration as opposed to exploitation from the recorded data, we must make several assumptions about their behaviour. Reinforcement learning provides a straightforward approach to modelling this type of decision-making.

***Calculation of the likelihood.*** The binary two-armed bandit task was simulated as described and the number of unsuccessful draws with an aversive outcome was recorded. The probability of receiving a certain number of painful stimuli in one game is dependent on the choices of the participant on one hand and the reward probabilities of each arm on the other hand. The latter are set in the experimental setup and thereby known. The former was modelled by a softmax algorithm


$$p(a_{j})=\frac{exp\left(\frac{Q(a_{j})}{\tau }\right)}{\sum_{i=1}^{k}exp\left(\frac{Q(a_{i})}{\tau }\right)},\;\text{\#}(14)$$

where the parameter τ determines the degree of exploration. A higher value of τ correlates to a higher degree of exploration. The Q-value is a weighted average of previous rewards


$$Q_{n}^{j}=Q_{n-1}^{j}+\alpha (R_{n-1}-Q_{n-1}^{j}),\;\text{\#}(15)$$

with *R _i_* representing the reward on trial *i*. The learning rate α was fixed at 0.1. The Q-values for both arms were initialised with *Q*_0_ = 0 and updated after each choice. This means that we need to consider not only the number of painful stimuli that were previously received when playing a certain arm, but also the order of these outcomes. In our case, the agent has six free choices per game. Therefore, there are seven possible outcomes, as the agent can receive between zero and six painful stimuli.

In each trial, participants choose between two arms, which we call *a* and *b*. The probabilities of choosing the respective arms are dependent on the previous choices and received rewards. Once a choice is made, there is a certain probability of receiving a painful stimulus that in our example is only dependent on the arm. In the case of a dynamic multi-armed bandit, this probability would vary with the trial number.

For each choice, there are four possible combinations of arm chosen and aversive or neutral reward. For six free choice trials there are a total of 4 ^6^ possible paths, or combinations of choices and outcomes. We can calculate the probability of each of these paths as a product of the probabilities of the choices and corresponding outcomes within this path. The probabilities for each choice can be calculated from ( 14).

To obtain the probability of each possible outcome measure, i.e. the number of aversive outcomes *n _a_* in a game, we iterate through all possible paths resulting in this outcome and sum them up. As we have seen in ( 14), this probability is dependent on the variable *τ*, which determines the degree of exploration in the behaviour. The iterations and calculations can be repeated for all possible outcomes *n _a_*, as well as a set of different *τ*, which will give us *P*( *y _i_* |τ). The likelihood for a collected dataset, *P*( *Y*|τ), can then be calculated according to ( 4). 

## Results

The use of the BFDA as a method for prospective design analysis allows us to consider a number of factors. However, it is essential to evaluate the algorithm estimating the latent variable as a preliminary step. Once the algorithm has been validated, it can be used for the BFDA, in which repeated sampling from a population is simulated and the planned analysis carried out on the sample. In this work we first examine the distribution of Bayes factors, which indicate whether there is evidence for or against a difference in population mean between groups. This analysis is carried out to determine possible effect sizes and the effect of experimental parameters, such as the number of participants, as well as the number of games per participant on the evidence. In the last step, we analyse potential errors when estimating a difference in means between groups and how they are affected by the aforementioned experimental parameters.

### Validation of the estimation algorithm

To validate the estimation algorithm, human choices on the multi-armed bandit task were simulated for a range of exploration parameters. Each simulated dataset consisted of a range of sample sizes between 10 and 58 in increments of 2 with *n*_games_= 150 games per ‘participant’. The individual exploration parameter for each simulated participant was drawn from a normal distribution with population mean *μ _τ_* ranging from 0.05 to 1 in increments of 0.05, and a standard deviation of *σ* = 0.02. The goal was to then estimate the population mean within the Bayesian framework as described.

To evaluate the accuracy of this estimation we can compare the point estimates calculated as stated in ( 5) with the true exploration parameters. Multiple regression analysis was used to investigate the correlation between the mean estimated exploration parameter with the sample size and the true population mean. The overall fit of the regression model was statistically significant ( *F*(3,496) = 136300, *p* < 0.001, *R* ^2^ = 0.999). The sample size and population mean, as well as their interaction were significant predictors of the mean estimations ( *t* = 7.246, *p* < 0.001; *t* = 265.589, *p* < 0.001; *t* = –17.442, *p* < 0.001). The mean squared estimation error (MSEE) was calculated and revealed strong negative correlations with the sample size across all simulated mean exploration parameters ( *r*(23) < -0.96, *p* < 0.001). The relationship between the MSEE and the sample size, as well as population mean, was analysed using multiple regression analysis. After visual inspection, the sample size was log-transformed and the population mean was exponentially transformed. The overall fit of the regression model was statistically significant ( *F*(3,496) = 7763, *p* < 0.001, *R* ^2^ = 0.979). The transformed sample size and mean, as well as their interaction, were significant predictors of the MSEE ( *t* = 9.284, *p* < 0.001; *t* = 126.156, *p* < 0.001; *t* = –96.491, *p* < 0.001). These results demonstrate that the algorithm is successful in estimating the mean exploration parameter within a group. It is also shown that the MSEE decreases with increasing sample size, which indicates that the estimations increase in accuracy with more participants. This is a crucial point that must be validated before proceeding with the analysis, as it forms the foundation for subsequent considerations, such as sample size analyses. 

In order to evaluate the accuracy of the estimations of a difference in mean between two populations, the estimated difference between the two populations ${\hat{\Delta \mu }}_{\text{τ}}$ was compared to the true difference Δ *μ*_τ_. 250 simulations were run for each combination of Δ *μ*_τ_ ranging from 0 to 1 in increments of 0.05 and sample size per group ranging from 10 to 60 in increments of 2. Multiple regression analysis was used to assess the relation between the mean estimations and the sample size, as well as the true difference in population mean. The overall fit of the regression model was statistically significant ( *F*(3,496) = 137900, *p* < 0.001, *R* ^2^ = 0.999). The difference in population mean as well as the interaction between the difference in mean and the sample size were significant predictors of the mean estimations ( *t* = 266.473, *p* < 0.001; *t* = –16.932, *p* < 0.001), while the sample size was not ( *t* = 0.522, *p* = 0.602). The MSEE was calculated for the simulated range of *n*. The relationship between the MSEE and the sample size, as well as the difference in means, was explored using multiple regression analysis. After visual inspection, the sample size was log-transformed and the difference in means was exponentially transformed. The overall fit of the regression model was statistically significant ( *F*(3,496) = 3506, *p* < 0.001, *R* ^2^ = 0.955). The transformed sample size and difference in means, as well as their interaction, were significant predictors of MSEE ( *t* = –24.571, *p* < 0.001; *t* = 54.985, *p* < 0.001; *t* = –42.632, *p* < 0.001). These results indicated that the algorithm is effective in estimating the difference in the random exploration parameter between two groups. It is also highlighted that the MSEE decreases with increasing sample size, indicating that the estimations become more precise with an increased number of participants. Having validated that the analysis can accurately estimate the population mean, as well as the difference in means between two groups, we can use it in combination with BFDA.

### Bayes factor design analysis

BFDA entails simulating behavioural data from repeated samples of a population. The analysis of each simulated sample follows the same procedure as the planned analysis of the real dataset. The experiment's design can then be evaluated by calculating the probability of the analysis supporting the null or alternative hypothesis, as well as the probabilities of errors, such as finding evidence in favour of the wrong hypothesis or overestimating the effect size. This analysis looks at the Bayes factor as a function of the difference in mean Δ *μ*_τ_, sample size *n* of each group and number of games per participant *n*_games_. In addition, the interplay between *n* and *n*_games_ was further investigated by considering the average length criterion for sample size determination.

**Figure 2.**
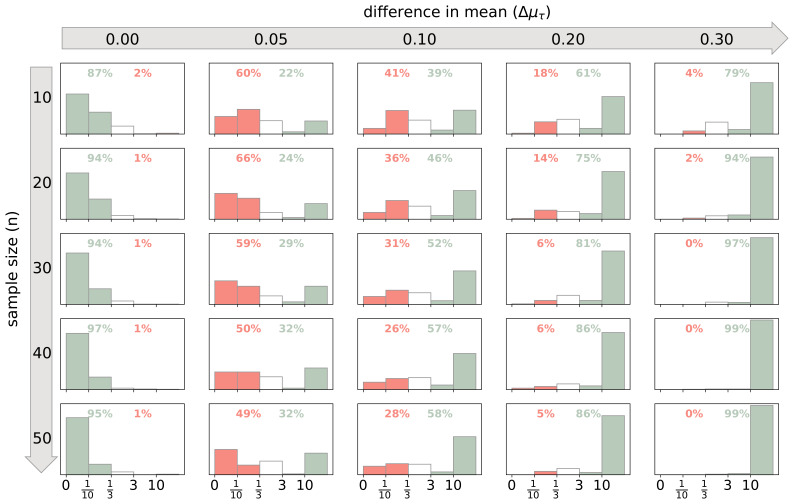
Probabilities of Bayes factors indicating different strengths of evidence. Data was simulated to test for a difference in the degree of exploration between two groups. For each combination of sample size of each group *n* and the difference in mean *Δμ*_τ_ 250 simulations were performed. The number of games per participant was *n _games_* = 150. The green bars show the relative frequency of a Bayes factor indicating at least moderate evidence in favour of the correct hypothesis, while the red bars show the relative frequency of at least moderate evidence in favour of the incorrect hypothesis. The cumulative probabilities for these instances are given as percentages. The white bars show the relative frequencies of anecdotal evidence for either hypothesis.

***Determining possible effect sizes.*** First, 250 simulations were run for each combination of the sample size of each group ranging from 10 to 60 in increments of 2 and difference in means ranging from 0 to 0.95 in increments of 0.05. The Bayes factor was calculated for each of these simulations. The relative frequencies of the Bayes factor *BF*_10_ indicating different strengths of evidence is shown in Figure 2 for representative values of Δ *μ*_τ_ and *n*. With an increase of Δ *μ*_τ_ and *n* the Bayes factors are more likely to support the correct hypothesis and the stronger the evidence in favour of the correct hypothesis.

**Figure 3.**
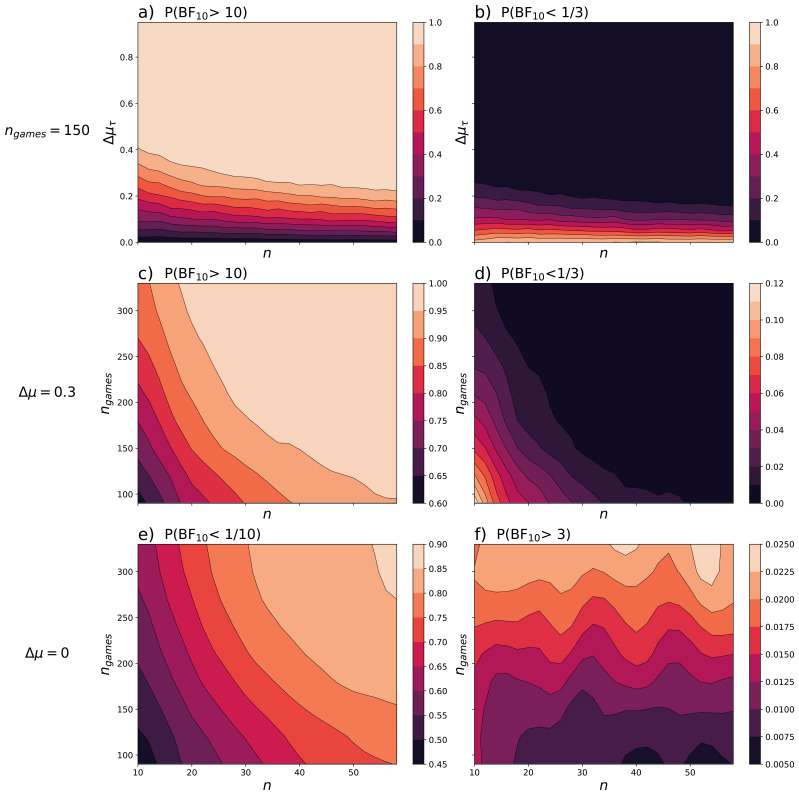
Probabilities of a Bayes factor indicating evidence in favour of the null and alternative hypothesis. Data was simulated to test for a difference in the degree of exploration between two groups. The contour plots display interpolations of the relative frequency of a Bayes factor indicating at least strong evidence in favour of the alternative hypothesis ( **a**, **c**, **f**) and of the null hypothesis ( **b**, **d**, **e**). The relative frequency was considered as a function of *n* and *Δμ _τ_* for *n _games_* = 150 ( **a**, **b**), and as a function of *n* and *n _games_* for the true differences of *Δμ _τ_* = 0.3 ( **c**, **d**) and *Δμ _τ_* = 0 ( **e**, **f**). The contour plots were smoothed with a Gaussian filter ( *a,b*: *σ* = 0.5; c, d: *σ* = 0.8; e, f: *σ* = 1.3).

The relative frequency of a Bayes factor higher than 10 is shown in Figure 3a for the simulated values of Δ *μ*_τ_ and *n*. This Bayes factor would indicate at least strong evidence for the alternative hypothesis, which for this example states that there is a difference in the exploration parameter between two groups. For a true difference in the mean degree of exploration *Δμ _τ_* ≠ 0, this probability of at least strong evidence supporting the alternative hypothesis would correspond to the power in frequentist statistics, assuming we reject the null hypothesis for a Bayes factor higher than 10.

Similarly, we can calculate the probability of receiving a Bayes factor *BF*_10_ smaller than $\frac{1}{3}$ which would indicate at least moderate evidence in favour of the null hypothesis ( Figure 3b). For Δ *μ _τ_* ≠ 0 this probability is the probability of wrongfully accepting the null hypothesis, which corresponds to the type II error. One way to visualize these probabilities is via a contour plot as shown in Figure 3. Figures 3a,b demonstrate the ability of the algorithm to distinguish a difference in the degree of exploration between two groups as a function of the actual difference and their sample size. As the sample size and true difference increase, the probability of finding strong evidence for the alternative hypothesis increases. These plots can help to determine the necessary difference in means to support the alternative hypothesis for a given sample size, or vice versa. If an approximation of the true difference in means is known, it is possible to estimate the sample size needed to demonstrate a difference in population means. However, this does not guarantee an accurate estimation of the difference but rather provides evidence for or against the null hypothesis.

Using a rough estimate of *μ _τ_* ≈ 0.1, we can infer from Figure 3a that about 40 participants per group would be needed to achieve an above 50% chance of the analysis yielding Bayes factors above 10. Adding more participants does not appear to have a substantial impact and only marginally improves the chances. For this exemplary sample size of *n* = 40 and an exploration parameter of *μ _τ_* = 0.1, the probability of obtaining a Bayes factor smaller than $\frac{1}{3}$ is around 26%. This means that there is a 26% likelihood of finding at least moderate evidence in favour of an incorrect hypothesis. On the other hand, if the null hypothesis were true ( *μ _τ_* = 0), we would expect a Bayes factor greater than 10 with a probability of 0.4%, indicating at least strong evidence in favour of the alternative hypothesis, and a Bayes factor less than $\frac{1}{3}$ with a probability of 96.8%, indicating at least moderate evidence in favour of the null hypothesis.

***Balancing the number of games per participant and sample size.*** When designing experiments, it is worth considering the balance between the number of participants and the number of trials each participant completes. The relative frequency of a Bayes factor higher than 10, *P*( *BF*_10_ > 10), was calculated across 500 simulations per combination of the sample size of each group *n* and the number of games per participant *n*_games_, as shown in Figure 3c. These calculations were carried out for a true difference in mean of Δ *μ _τ_* = 0.3. This value was chosen arbitrarily to demonstrate how the number of participants and the number of trials affect the evidence in favour of a difference in means. The simulations showed an increase in the relative frequency of a Bayes factor *BF*_10_ higher than 10 with an increase in the sample size and number of individual trials. This is supported by positive correlations between *P*( *BF*_10_ > 10) with *n* and *n*_games_ ( *r _s_* (23) = 0.74, *p* < 0.001; *r _s_* (7) = 0.62, *p* < 0.001) and negative correlations between *P*( *BF*_10_ < 1/10) with *n* and *n*_games_ ( *r _s_* (23) = –0.49, *p* < 0.001, *r _s_* (7) = –0.31, *p* < 0.001).

The appropriate balance between *n*_games_ and *n* is dependent to external factors. For instance, to achieve a probability of at least 90% for obtaining a Bayes factor greater than 10, we could consider a sample size of 20 participants per group each completing 200 games. Alternatively, a sample size of 30, with each participant completing 130 games could be implemented ( Figure 3c), resulting in a smaller total amount of games played. For both options the probability of wrongfully supporting the null hypothesis is below 2% ( Figure 3d). Assuming all participants complete the same number of trials per hour, the second option would result in less expenses for participants paid at an hourly rate. This would be suitable for an online study, with no additional costs per participant. However, if the study incurs additional costs per participant, the first option may be preferable. When selecting an appropriate sample size, it is important to also ensure that the chosen parameters provide Bayes factors to favour the null hypothesis if it is true. This can be conducted by repeating the above simulations for a true difference of Δ *μ _τ_* = 0 as shown in Figures 3e,f.

**Figure 4.**
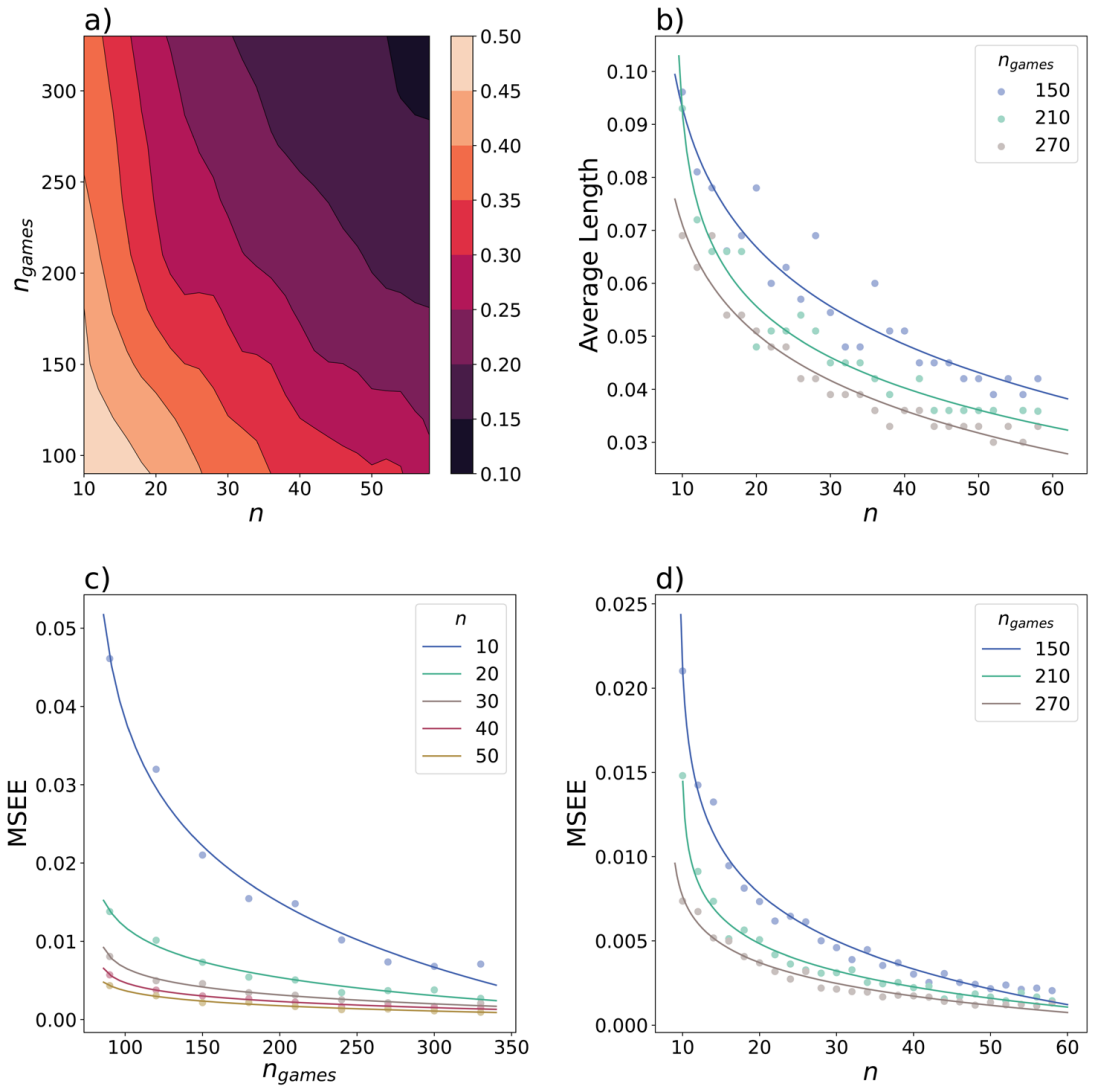
Further considerations for an optimal balance between number of games per participant and sample size. Data was simulated to test for a difference in the degree of exploration between two groups with a true difference of *Δμ _τ_* = 0.3. For each combination of sample size of each group *n* and the number of games per participant *n _games_*, 500 simulations were performed. ( **a**) The magnitude error was calculated for all simulations resulting in a Bayes factor greater than 10. The contour plot displays an interpolation of the relative frequencies of a magnitude error exceeding 10%, smoothed with a Gaussian filter ( *σ*= 0.9). ( **b**) The 95% HPDI of the posterior was determined as a function of *n* and *n _games_*, and the average length was calculated. The mean squared estimation errors were calculated and sample values are shown to illustrate the relationship between the mean squared estimation error, *n _games_* and *n* ( **c**, **d**).

To gain an understanding of how the Bayes factor relates to the true estimate of the difference in mean, we can determine the magnitude error for those simulations that result in a Bayes factor greater than 10. The magnitude error is calculated by dividing the difference between the estimated value and the true difference in mean by the true difference in mean. This reflects an overestimation of the true effect size in statistically significant results ( Gelman & Carlin, 2014). The probability of a magnitude error exceeding 10% decreases with an increase of *n* and *n _games_* ( Figure 4a). This may seem trivial, as it is expected that our calculations become more accurate when more data is available. However, the probability of overestimating the effect is an important aspect to consider in both prospective and retrospective design analysis as the ultimate goal is to obtain a realistic estimate of the effect size. Simulations as carried out here can aid in choosing optimal values of *n* and *n _games_* to minimize the probability of errors.

To illustrate further, we can consider our example from the previous section, where we compared *n* = 30 with *n*_games_ = 130 to *n* = 20 with *n*_games_ = 200. The probability of obtaining a Bayes factor greater than 10 was 90% for both options. To decide between the two options, we can consider the magnitude error. The first option has a 35% probability of overestimating the effect by at least 10% for all significant results. For the latter, this probability is between 30% and 35%. Therefore, the latter option is less likely to overestimate the true effect size, although these probabilities are still relatively high.

As another measure of the accuracy of our estimation of the effect size, the average length of the *HPDI*_.95_ was calculated from the simulated data for each combination of sample size ranging from 10 to 58 and *n*_games_ ranging from 90 to 330 of which representative values are shown in Figure 4b. The average length decreased with increasing *n* and *n*_games_, which corresponds to a narrower posterior distribution and therefore a higher certainty about the value of the estimated parameter. However, the average length does not provide any information regarding the location of the highest probability densities. Consequently, our simulations may result in a highly narrow posterior distribution that is centred around a wrong estimation. To evaluate the validity of our estimations, the MSEE was calculated, which showed a decrease as *n* and *n*_games_ increase ( Figure 4c,d). If both the error and average length of the HPDI are decreasing with *n*, the estimations are increasingly accurate. 

## Discussion

Bayesian statistics offer a robust framework for parameter estimation and hypothesis testing across a range of problems ( Ashby, 2006; van de Schoot *et al.*, 2017), and are therefore a valuable alternative to frequentist methods. However, in Bayesian statistics, prospective design analyses are less common compared to their frequentist counterpart, and protocols for effective experimental designs and testing methods need to be established. In this work, we used BFDA to analyse the effect of sample size, number of games per participant, and effect size on the probability of obtaining significant evidence to support a null or alternative hypothesis, and the probabilities of incorrectly supporting either hypothesis. This furthers previous work on BFDA ( Schönbrodt & Wagenmakers, 2018; Stefan *et al.*, 2019; Stefan *et al.*, 2024) by incorporating additional aspects of experimental design which are critical in behavioural sciences. Additionally, we contribute to existing knowledge by integrating latent variable analysis into BFDA, as demonstrated by a practical example using a multi-armed bandit task. The prospective design analysis was expanded by examining the average length criterion for studies investigating precise estimates of the difference between groups.

The example of a multi-armed bandit (MAB) task with prior information was used to examine latent variables in relation to BFDA. Previous studies have focused on inferring the degree of random exploration from behavioural outcomes in this task ( Mizell *et al.*, 2024; Somerville *et al.*, 2017; Waltz *et al.*, 2020; Wilson *et al.*, 2021; Wilson *et al.*, 2014), with significant differences in random exploration being identified between certain populations ( Mizell *et al.*, 2024) and not others ( Mizell *et al.*, 2024; Waltz *et al.*, 2020). These studies employed frequentist methods, which do not provide evidence in favour of a null hypothesis. Therefore, there is no evidence supporting the absence of a difference between the groups. Our study validated a Bayesian analysis for the MAB with prior information using Bayes factors and estimations of the difference. We found that it can be challenging to determine the difference in means between two groups. Large sample sizes are needed for a strong probability of detecting evidence in favour of difference, where present. If there is strong evidence supporting the alternative hypothesis, the probability of overestimating the difference in means is relatively high. In the example we used an uninformative prior, but depending on the experiment and existing literature, this should be adjusted. A well-informed prior can lead to higher probabilities of detecting a difference between groups.

Optimizing experimental parameters can increase the likelihood of finding evidence supporting either null or alternative hypothesis while reducing the likelihood of overestimating the true effect. Previous research has investigated the most efficient design for MAB tasks by optimising a utility function ( Valentin *et al.*, 2024; Zhang & Lee, 2010). This function aims to maximise the information gain of each design ( Ryan *et al.*, 2016). However, it is important to consider the probability of errors, such as overestimating effects, as well as resource considerations, such as cost and space, as described in our work.

We demonstrated that that the probability of a Bayes factor indicating strong evidence highly depends on the number of games each participant completes. The analyses indicate that, in certain situations, it may be more efficient to increase the number of trials per participant than to increase the number of participants. While this might seem intuitive, as a higher number of games per participant translated into more data, it is not traditionally included in power analyses. These results add to previous research on frequentist methods ( Baker *et al.*, 2021; Rouder & Haaf, 2018), which suggest the inclusion of this parameter in design analyses.

Considerations about parameters such as sample size and the number of games per participant can be extended beyond the given example to studies measuring, for example, reaction times, EEG, MEG, or fMRI ( Baker *et al.*, 2021; Lorenz *et al.*, 2017). If these analyses are considering latent variables, a suitable model of how this latent variable affects the outcome measure is needed. Previous studies in neuroscience have combined latent variable modelling with various methods including EEG ( Ghaderi-Kangavari *et al.*, 2022; Li *et al.*, 2020), MRI ( Cooper *et al.*, 2019; Lahey *et al.*, 2012; Tien *et al.*, 1996), reaction times ( Jaffe *et al.*, 2023) and cognitive tasks ( Decker *et al.*, 2014; Jaffe *et al.*, 2023). If a suitable model has not been established, a pilot study may provide one that can be used for the prospective design analysis.

The example used in this study is a simple version of an exploration task, using binary outcomes in a 2-choice paradigm. There are many different ways of targeting exploration, including using continuous outcomes (which is less straightforward for non-numerical outcomes such as pain) non-stationary paradigms (in which the outcome probabilities change over time), and larger numbers of options (e.g. 4 bandits). Another complexity is that humans use more than one type of exploration strategy ( Seymour *et al.*, 2012), indeed the horizon task here was explicitly designed to explore so-called ‘directed’ exploration, which is proposed to operate over-and-above random exploration, according to estimates of outcome uncertainty ( Wilson *et al.*, 2021; Wilson *et al.*, 2014). The approach we show here can be equally applied to these more complex paradigms and analyses (i.e. computational models). Here we restricted ourselves to the simplest design for clarity. 

In terms of potential limitations, the model used could be improved by modelling changes in exploration throughout a game or by extending the in-population variability to the learning rate. For the BFDA, rather than recording the total number of aversive outcomes for each game, an alternative approach would be to record the choice made in each trial and compute a likelihood function for choosing the respective arm for all possible Q-values for each trial. However, the resulting probabilities in the present approach regarding possible errors are still valuable, as they estimate appropriate sample sizes and number of games per participant. The dependence on an accurate model is a drawback of using BFDA with latent variables and restricts its uses as a prospective design analysis in cases where a model is missing. However, once a model is established, it can still be used as a retrospective design analysis to provide information on the probabilities of errors.

We argue that design analyses could benefit from calculating not only the optimal sample size and number of games per participant, but also from extending the analysis to other parameters. BFDA enables us to examine the effect of parameters such as the prior distribution or, more specific to the simulated task, the probabilities of aversive outcomes, the number of free choice trials, or the difference between continuous and binary outcomes. These considerations can help us make the best use of available resources.

## Data Availability

Zenodo: sarah407/BFDA_Multi-Armed-Bandit: v1.0, https://doi.org/10.5281/zenodo.12507303 ( Schreiber, 2024). This project contains the simulated data. Data are available under the terms of the Creative Commons Attribution 4.0 International license (CC-BY 4.0).

## Code availability

**Source code available from:** https://github.com/sarah407/BFDA_Multi-Armed-Bandit

**Archived source code at time of publication:** https://doi.org/10.5281/zenodo.12507303 ( Schreiber, 2024).

**License:** Code are available under the terms of the Creative Commons Attribution 4.0 International license (CC-BY 4.0).
