# Supplementary material for: Enhancing experimental design through Bayes factor design analysis: insights from multi-armed bandit tasks
Source: Wellcome Open Res. 2026 May 7;9:423. Originally published 2024 Aug 1. [Version 2] doi: 10.12688/wellcomeopenres.22288.2 (PMC13241856; doi:10.12688/wellcomeopenres.22288.2)
Supplement: Peer review history

# Reviewer report: Reviewer response for version 2

**DOI:** 10.21956/wellcomeopenres.28844.r157351

**Published:** 2026-06-13

**Authors:** Alokesh Manna

**Affiliations:** 1 University of Connecticut, Storrs, USA

**Summary**

 The paper is primarily a tutorial illustration rather than a methodological advance. The core tools — BFDA, softmax RL, HPDI, and Bayesian parameter estimation — are all already in place. The novelty is their combination and the demonstration that latent-variable BFDA is feasible and informative for behavioral experiment design.

**Questions**

 Here are a few comments:

 a) In the method section, a clear parameter space, sample space, and data-generating space should be mentioned. Perhaps the authors intend to write for a very broader scientific community, even those who have little knowledge of Bayesian statistics. However, to be mathematically sound is fundamental. What is the data? What is the dimension of the parameter space? etc. must be clarified even before jumping to the method section.

 b) All the equations must be numbered. Otherwise, it is hard to point out which portion I want more clarification.

 c) "From the posterior, a point estimate of θ can be calculated using" — the equation has a trivial mistake for not writing the prior as a multiplication.

 d) Are you trying to define the Bayes factor anew? What are Y₁, Y₂?

 e) Before defining the Bayes factor, you must define the angle of hypothesis testing. Otherwise, the story is not clear.

 f)  A gentle clarification of p-value and HPD is highly recommended. It should be clarified where p-value gets stuck. How does Bayesian decision science help to clarify that?

 g) "Calculation of the likelihood" — the authors should use LaTeX for writing the subscripts. Most of the components are undefined.

 h) Organizing the analysis around BF > 10 and BF < 1/3 risks reproducing frequentist threshold logic — the very criticism Bayesian approaches are meant to address.

 i) A fundamental assumption underlying the proposed BFDA framework is that observations are independently and identically distributed (i.i.d.) across participants and games. This assumption is violated in many practically important settings included in references.

 In such settings, the effective sample size is strictly less than the nominal n, rendering the BFDA power curves over n potentially misleading. The authors are encouraged to explicitly state the i.i.d. assumption as a formal condition, discuss how the framework breaks down when it fails, and identify dependent-data extensions as a concrete direction for future research indicating the referenced papers and a few other relevant works where i.i.d. assumptions can be violated.

 j) The authors should mention that in probability theory, de Finetti's theorem states that exchangeable observations are conditionally independent relative to some latent variable. It should be clarified how this works in the Bayesian settings and helps to resolve the non-i.i.d. issue.

Is the rationale for developing the new method (or application) clearly explained?

Yes

Is the description of the method technically sound?

Partly

Are the conclusions about the method and its performance adequately supported by the findings presented in the article?

Yes

If any results are presented, are all the source data underlying the results available to ensure full reproducibility?

Yes

Are sufficient details provided to allow replication of the method development and its use by others?

Partly

Reviewer Expertise:

Bayesian analysis

I confirm that I have read this submission and believe that I have an appropriate level of expertise to confirm that it is of an acceptable scientific standard, however I have significant reservations, as outlined above.

## References

[1] : Time Series: Theory and Methods. 10.1007/978-1-4419-0320-4 10.1007/978-1-4419-0320-4

[2] : Bayesian variable selection for matrix autoregressive models. *Statistics and Computing* .2024;34(2) : 10.1007/s11222-024-10402-y 10.1007/s11222-024-10402-y

[3] : Statistics for Spatial Data. 10.1002/9781119115151 10.1002/9781119115151

[4] : Linear Mixed Models for Longitudinal Data. 10.1007/978-1-4419-0300-6 10.1007/978-1-4419-0300-6

# Reviewer report: Reviewer response for version 2

**DOI:** 10.21956/wellcomeopenres.28844.r155003

**Published:** 2026-06-05

**Authors:** Jessica Schaaf

**Affiliations:** 1 Radboudumc (Ringgold ID: 6034), Nijmegen, Gelderland, The Netherlands; 2 Medical Neuroscience, adboud University Donders Institute for Brain Cognition and Behaviour (Ringgold ID: 198328), Nijmegen, Gelderland, The Netherlands

I thank the authors for their revisions acknowledging the boundaries of their method and referring to additional material where necessary. I do have some remaining comments.

 In response to point 1, you have extended the README file in the Github repository. Although it already helps to know what every script does and what it outputs, the README file still doesn’t state *why *I would run a given script and based on which output I should conclude which design to use. I think it would be a shame if researchers do not apply your elegant method because it requires too much cognitive effort.

 In response to point 7, you have added the coding of outcomes. I understand the combination of the coding of outcomes and the initialization of Q-values doesn’t matter in simulation studies as long as you simulate and refit with the same coding. However, in empirical data, it is common to initialize the Q-value in between the possible outcomes (in your case -0.5). Otherwise you assume participants are biased towards expecting no aversive outcome a priori, which affects learning rate estimation. It would be good to explicitly state the rationale for choosing this Q-value initialization (or simply state it was an arbitrary choice). 

 In response to point 8, you adjusted Figures 3 and 4. Really helpful. I don't see green circles in Figure 3c and d though.

Is the rationale for developing the new method (or application) clearly explained?

Yes

Is the description of the method technically sound?

Yes

Are the conclusions about the method and its performance adequately supported by the findings presented in the article?

Yes

If any results are presented, are all the source data underlying the results available to ensure full reproducibility?

Yes

Are sufficient details provided to allow replication of the method development and its use by others?

Partly

Reviewer Expertise:

Bayesian computational modeling; reinforcement learning; decision making; cognitive development

I confirm that I have read this submission and believe that I have an appropriate level of expertise to confirm that it is of an acceptable scientific standard.

# Reviewer report: Reviewer response for version 2

**DOI:** 10.21956/wellcomeopenres.28844.r155582

**Published:** 2026-06-05

**Authors:** Tommaso Costa

**Affiliations:** 1 University of Turin, Turin, Italy

This manuscript presents a tutorial-style methodological framework combining Bayes factor design analysis (BFDA) with latent variable modelling in the context of multi-armed bandit (MAB) tasks. The article addresses an important and timely problem in cognitive neuroscience and behavioural science, namely how to prospectively optimise experimental designs when the quantities of interest are latent computational parameters rather than directly observed variables.

 The manuscript is clearly motivated and generally well written. A major strength of the work is the integration of BFDA with computational modelling approaches commonly used in reinforcement learning and decision neuroscience. The paper also contributes by explicitly considering probabilities of inferential errors, uncertainty estimates, and practical trade-offs between sample size and number of trials per participant. The open availability of code and data further strengthens the transparency and reproducibility of the work.

 Overall, I believe the article makes a useful methodological contribution and is suitable for indexing after revision. My main concerns relate not to the general validity of the framework, but rather to the interpretation, generalisability, and robustness of the presented implementation.

 Major comments

1. Parameter identifiability and simplicity of the computational model

One important limitation is that the current demonstrations rely on a relatively simplified computational model. The analyses focus primarily on a single softmax exploration parameter τ with a fixed learning rate. While this simplification is understandable for tutorial purposes, it substantially reduces the complexity typically encountered in realistic reinforcement-learning analyses.

 In many practical bandit paradigms, exploration behaviour depends on multiple partially correlated latent processes, including directed exploration, uncertainty bonuses, perseveration, or variable learning rates. Under these conditions, parameter identifiability can become substantially more difficult.

 The manuscript demonstrates encouraging recovery properties using MSEE analyses and regression summaries, but additional discussion of parameter identifiability would strengthen the methodological contribution. In particular, the authors should discuss more explicitly:

- parameter trade-offs,
- posterior parameter correlations,
- and the extent to which the present conclusions are expected to generalise to richer computational models.

Although full additional analyses may not be strictly necessary for publication, a more explicit discussion of these limitations is important to ensure appropriate interpretation of the framework.

1. Prior sensitivity analysis

The manuscript correctly acknowledges the importance of prior specification in Bayesian inference. However, given that Bayes factors can be highly sensitive to prior assumptions, it is somewhat surprising that the paper does not include a more systematic prior sensitivity analysis.

 The current demonstrations rely primarily on uniform priors for illustrative purposes. While this is acceptable for a tutorial paper, the manuscript would benefit from either:

- a brief sensitivity analysis across different plausible priors,
  or
- a more detailed discussion of how prior specification may affect BFDA conclusions in practical applications.

This point is particularly important because BFDA is intended as a prospective design tool, and design recommendations may depend substantially on prior assumptions.

1. Interpretation of Bayes factor thresholds

The manuscript appropriately notes that Bayes factors are continuous measures of evidence and should not be interpreted simply as Bayesian analogues of p-values. However, much of the analysis is nevertheless organised around threshold-based interpretations such as BF > 10 or BF < 1/3.

 While this approach is understandable for pragmatic design-analysis purposes, the manuscript would benefit from a slightly deeper discussion clarifying:

- the pragmatic role of these thresholds,
- their relationship to decision-making,
- and the limitations of threshold-based evidence categorisation.

At present, the framework risks partially reproducing a threshold-centred logic analogous to traditional significance testing. Clarifying this distinction would strengthen the conceptual framing of the paper.

1. Generalisability and scalability

The manuscript demonstrates the framework using a relatively simple binary two-armed bandit task. Although the authors discuss possible extensions to more complex paradigms, the practical scalability of the method remains somewhat unclear.

 It would be useful for the manuscript to elaborate further on:

- computational burden,
- scalability to hierarchical models with multiple latent parameters,
- runtime considerations,
- and applicability to more complex task structures.

This additional discussion would improve the translational relevance of the framework for researchers working with more realistic behavioural and neurocomputational models.

 Minor comments

1. Some notation could be clarified further, particularly regarding the distinction between participant-level latent parameters and population-level parameters.
2. A brief explanation of why a fixed-n BFDA framework was prioritised over sequential BFDA approaches would be useful.
3. A final language-polishing pass would improve readability in some sections.
4. Some figures are visually dense and may benefit from slightly larger labels or simplified visual presentation.

Overall assessment

 This is a thoughtful and methodologically valuable contribution that extends BFDA into an important area of computational cognitive neuroscience. The manuscript is transparent, technically competent, and addresses practically relevant design problems that are often neglected in behavioural research.

 The integration of latent-variable modelling with prospective Bayesian design analysis is particularly valuable, and the emphasis on uncertainty, inferential errors, and design trade-offs is a major strength of the paper.

 I recommend approval with reservations. The points above should be addressed to strengthen the robustness,interpretability, and generalisability of the proposed framework.

Is the rationale for developing the new method (or application) clearly explained?

Yes

Is the description of the method technically sound?

Partly

Are the conclusions about the method and its performance adequately supported by the findings presented in the article?

Partly

If any results are presented, are all the source data underlying the results available to ensure full reproducibility?

Yes

Are sufficient details provided to allow replication of the method development and its use by others?

Yes

Reviewer Expertise:

Bayesian Statistics; Research Methodology; Cognitive Neuroscience

I confirm that I have read this submission and believe that I have an appropriate level of expertise to confirm that it is of an acceptable scientific standard, however I have significant reservations, as outlined above.

# Reviewer report: Reviewer response for version 1

**DOI:** 10.21956/wellcomeopenres.24565.r112332

**Published:** 2024-12-16

**Authors:** Jessica Schaaf

**Affiliations:** 1 Radboudumc (Ringgold ID: 6034), Nijmegen, Gelderland, The Netherlands; 2 Medical Neuroscience, adboud University Donders Institute for Brain Cognition and Behaviour (Ringgold ID: 198328), Nijmegen, Gelderland, The Netherlands

The authors provide a Bayesian approach to do prospective planning of experimental studies including computational modeling. They illustrate their approach for a horizon task and do extensive simulations showing how to choose a sample size and the number of games per participant for different scenarios. I personally enjoyed reading the paper and think it provides a clear story on how to use Bayesian methods to optimize experimental research.

 Below I provide some suggestions on how to improve the applicability and clarity of the method. As these are mostly clarifications and extensions, I consider them minor revisions.

1. My most important point is the correspondence between the target audience and the information provided. I assume the paper is meant for the applied researcher wishing to use the proposed method to plan their experiment. I applaud you from sharing your code, but I would not know where to start. Which considerations do I have to make *before *performing the simulations? What kind of range would I have to simulate? Which script(s) do I need to adjust? What are the software requirements for using the code? It would really help to have a step-by-step starters guide, either by upping the tutorial element of your paper or by providing a guide accompanying your code base.
2. One advantage of the two-armed experiment with fixed horizon is that it is fairly easy to map all possible choice and outcome patterns, and thus their likelihoods. Although you state that “the approach we show here can be equally applied to these more complex paradigms and analyses (i.e. computational models)”, I struggle to imagine how feasible the approach is in such cases as the number of possible patterns increases exponentially with more choice options and more trials. Please elaborate.
3. Somewhat relatedly, in the discussion you state that “the dependence on an accurate model is a drawback of using BFDA with latent variables and restricts its uses as a prospective design analysis in cases where a model is missing.” Although this is true, I wonder how this affects the applicability of your method. Say I want to test which model in a model set fits my data best (as is recommended practice, see e.g., Wilson & Collins, 2019; Van den Bos et al., 2018). Would this mean that I have to do a BFDA for all my competing models? And how do I then decide which sample size and number of games per participant to use?
4. Bayes Factor rely heavily on the chosen prior. Although you mention this yourselves in the method (“The chosen prior can heavily influence the outcome of an analysis and should therefore be chosen carefully, as it will bias estimation.”) and discussion (“In the example we used an uninformative prior, but depending on the experiment and existing literature, this should be adjusted. A well-informed prior can lead to higher probabilities of detecting a difference between groups.”), I miss explanation on how it exactly does so. It would be even better to also explicate considerations with respect to prospective planning.
5. In general, I find the paper clearly written. Yet, I got lost in the method section. Using a numerical example alongside the formulas would make the method more graspable.
6. Thank you for mentioning how you initialized Q-values (it is important!). Please also add how you coded rewards enabling readers to assess the accuracy of the initialization.
7. I really like the numerical examples (i.e., the combination of sample size, number of games, and probability of obtaining a certain BF) you used to illustrate the results and the beautiful plots. Yet, I struggled to connect the two. To guide readers, I think it would help to, for example, mark in the plot where the values in the text came from.

Is the rationale for developing the new method (or application) clearly explained?

Yes

Is the description of the method technically sound?

Yes

Are the conclusions about the method and its performance adequately supported by the findings presented in the article?

Yes

If any results are presented, are all the source data underlying the results available to ensure full reproducibility?

Yes

Are sufficient details provided to allow replication of the method development and its use by others?

Partly

Reviewer Expertise:

Bayesian computational modeling; reinforcement learning; decision making; cognitive development

I confirm that I have read this submission and believe that I have an appropriate level of expertise to confirm that it is of an acceptable scientific standard, however I have significant reservations, as outlined above.

## References

[1] : Ten simple rules for the computational modeling of behavioral data. 10.7554/eLife.49547 10.7554/eLife.49547 PMC687930331769410

[2] : Computational neuroscience across the lifespan: Promises and pitfalls. 10.1016/j.dcn.2017.09.008 10.1016/j.dcn.2017.09.008 PMC591650229066078

# Response

**Published:** 2026-04-25

**Authors:** Sarah Schreiber

**Affiliations:** University of Oxford, Oxford, England, UK

*The authors provide a Bayesian approach to do prospective planning of experimental studies including computational modeling. They illustrate their approach for a horizon task and do extensive simulations showing how to choose a sample size and the number of games per participant for different scenarios. I personally enjoyed reading the paper and think it provides a clear story on how to use Bayesian methods to optimize experimental research.*  

**Response**: We are thankful to Reviewer 3 for a generally positive evaluation of our work.

*Point 1 & 2.*

*My most important point is the correspondence between the target audience and the information provided. I assume the paper is meant for the applied researcher wishing to use the proposed method to plan their experiment. I applaud you from sharing your code, but I would not know where to start. Which considerations do I have to make before performing the simulations? What kind of range would I have to simulate? Which script(s) do I need to adjust? What are the software requirements for using the code? It would really help to have a step-by-step starters guide, either by upping the tutorial element of your paper or by providing a guide accompanying your code base.*  

**Response**: We thank the reviewer for this helpful comment. We agree that a step-by-step guide would make the manuscript more accessible. However, including a detailed tutorial within the manuscript itself would substantially increase its length and risk overwhelming readers with implementation details. Therefore, to address this concern, we have expanded the documentation in the GitHub repository (https://github.com/sarah407/BFDA_Multi-Armed-Bandit). Specifically, we have added clearer descriptions of the analysis scripts, outlined the software requirements, and included a conceptual checklist to guide users in setting up and running their own simulations. We have also provided references to additional resources for readers seeking more detailed guidance.

*Point 3.*

*One advantage of the two-armed experiment with fixed horizon is that it is fairly easy to map all possible choice and outcome patterns, and thus their likelihoods. Although you state that “the approach we show here can be equally applied to these more complex paradigms and analyses (i.e. computational models)”, I struggle to imagine how feasible the approach is in such cases as the number of possible patterns increases exponentially with more choice options and more trials. Please elaborate.*  

**Response**: We thank the reviewer for this important point. We agree that as task complexity increases (e.g., more options, longer horizons, or latent-variable models), the number of possible choice-outcome patterns grows rapidly, making exhaustive likelihood mapping computationally demanding. Our intention was to highlight that the general simulation-based framework can, in principle, be extended to more complex paradigms, such as MAB with more arms, dynamically changing reward probabilities, or hierarchical modelling, but only with careful consideration of computational constraints. In such cases, researchers would typically restrict the parameter space to theoretically relevant ranges, focus on key parameters, and limit the sample size grid. We have clarified in the manuscript that applying the approach to more complex designs requires explicit attention to runtime and scalability.

**Revised Section:** **“**The approach we show here can be equally applied to these more complex paradigms and analyses (i.e. computational models) with consideration of runtime and scalability. In general, simulation ranges should focus on values that are theoretically meaningful, rather than the broader ranges used here for demonstration purposes. For highly complex designs, approximate methods may be necessary, and efficient implementation (e.g. vectorisation, parallel computing, or cluster use) becomes increasingly important. **”**

*Point 4.*

*Somewhat relatedly, in the discussion you state that “the dependence on an accurate model is a drawback of using BFDA with latent variables and restricts its uses as a prospective design analysis in cases where a model is missing.” Although this is true, I wonder how this affects the applicability of your method. Say I want to test which model in a model set fits my data best (as is recommended practice, see e.g., Wilson & Collins, 2019; Van den Bos et al., 2018). Would this mean that I have to do a BFDA for all my competing models? And how do I then decide which sample size and number of games per participant to use?*

**Response**: We agree that this increases the complexity of the approach rather than fundamentally limiting it. Model comparison remains possible, but it requires additional simulation steps and clearly defined decision criteria. We have elaborated on this scenario in the revised manuscript to clarify how it can be implemented in practice.

**Revised Section:** **“**However, if the goal is to determine which model in a candidate set best explains the data, BFDA can be extended to compare models directly. The Bayes factor quantifies the relative evidence between two models ( , ) . One approach would be to simulate data under each candidate model and examine, across sample sizes and other design parameters, when the Bayes factor reliably favours the true generative model. However, this procedure becomes increasingly complex as the number of competing models grows.”

*Point 5.*

*Bayes Factor rely heavily on the chosen prior. Although you mention this yourselves in the method (“The chosen prior can heavily influence the outcome of an analysis and should therefore be chosen carefully, as it will bias estimation.”) and discussion (“In the example we used an uninformative prior, but depending on the experiment and existing literature, this should be adjusted. A well-informed prior can lead to higher probabilities of detecting a difference between groups.”), I miss explanation on how it exactly does so. It would be even better to also explicate considerations with respect to prospective planning. *  

**Response:** While an extensive treatment and comparison of prior specification is beyond the scope of this tutorial, we have added references to relevant literature to provide readers with further guidance.  

**Revised Section: ** “The chosen prior can heavily influence the outcome of an analysis and should therefore be chosen carefully, as it will bias the estimation. When prior knowledge about the underlying distribution is limited, researchers often employ uninformative or weakly informative priors, which can nevertheless reflect plausible bonds for the parameters. Given the subjective nature of priors, their selection should be transparently reported and sufficiently motivated. In addition, sensitivity analyses can be conducted to evaluate the robustness of results with respect to the chosen prior. For further discussions on priors, refer to further literature such as Gelman (2006), Van Dongen (2006), or Stefan (2019).”  

*Point 6.*

*In general, I find the paper clearly written. Yet, I got lost in the method section. Using a numerical example alongside the formulas would make the method more graspable.*

**Response**: We thank the reviewer for this suggestion. We agree that numerical examples can aid understanding, particularly in tutorial-style presentations. However, providing worked numerical examples for all formal expressions would require substantial restructuring of the Methods section and would considerably increase its length. We have revised the Methods section to improve clarity and readability, including clearer transitions and additional explanatory text where appropriate. We also provide references to established textbooks and methodological resources for readers seeking more step-by-step or example-based guidance.

**Added Section:** ***“Bayesian statistics.*** We begin by outlining the central concepts and key formulas of Bayesian statistics. Readers seeking more detailed explanations, formal derivations, and worked examples are referred to Kruschke (2014) and Hudson (2021).”  

*Point 7.*

*Thank you for mentioning how you initialized Q-values (it is important!). Please also add how you coded rewards enabling readers to assess the accuracy of the initialization.*

**Response**: We thank the reviewer for this comment and have adjusted the text accordingly.  

**Revised Section:** **“**In the computational modelling of these outcomes, each outcome must be assigned a numerical value. Here, the outcome of each choice was coded as 0 or -1, where -1 represents an aversive outcome. **”**  

*Point 8.*

*I really like the numerical examples (i.e., the combination of sample size, number of games, and probability of obtaining a certain BF) you used to illustrate the results and the beautiful plots. Yet, I struggled to connect the two. To guide readers, I think it would help to, for example, mark in the plot where the values in the text came from.*  

**Response**: We thank the reviewer for this helpful suggestion. To improve clarity and better connect the numerical examples in the text with the corresponding figures, we have revised the figures 3 and 4 to explicitly mark the parameter combinations referenced in the manuscript (e.g., specific sample sizes and numbers of games per participant).

**Revised Section:** “Using a rough estimate of *μ _τ_* ≈ 0.1, we can infer from Figure 3a that about 40 participants per group would be needed to achieve an above 50% chance of the analysis yielding Bayes factors above 10, highlighted with a white circle.” “The exact percentages reported here are obtained directly from our simulations, while the broader probability regions are illustrated in the contour plot (Figure 3).” “Alternatively, a sample size of 30, with each participant completing 130 games could be implemented (Figure 3c, green circles), resulting in a smaller total amount of games played. For both options the probability of wrongfully supporting the null hypothesis is below 2% (Figure 3d, green circles).” “To illustrate further, we can consider our example from the previous section, where we compared *n* = 30 with *n*_games_ = 130 to *n* = 20 with *n*_games_ = 200. The probability of obtaining a Bayes factor greater than 10 was 90% for both options. To decide between the two options, we can consider the magnitude error. The first option has close to a 35% probability of overestimating the effect by at least 10% for all significant results. For the latter, this probability is between 30% and 35% (Figure 4a, green circles). Therefore, the latter option is less likely to overestimate the true effect size, although these probabilities are still relatively high.”

# Reviewer report: Reviewer response for version 1

**DOI:** 10.21956/wellcomeopenres.24565.r95853

**Published:** 2024-11-26

**Authors:** Dominik Bach

**Affiliations:** 1 University of Bonn, Bonn, Germany

Schreiber et al. give a tutorial-style introduction into, and worked example of, the established framework of Bayes Factor Design Analysis, applied in the context of a multi-armed bandit task. No new method is developed. Thus, the original scientific contribution appears relatively small, but the worked example provides a welcome illustration of this (suite of) method(s) for students of the field. My major concern is that two assumptions of the method are not sufficiently discussed; there are also a couple of minor technical concerns.

 1. Any results hinge on the assumption of a specific data-generating process. If the true data-generating process (in a future experiment) is not the same as the one assumed in the simulation, then results such as required group size etc. will be misleading. The authors might want to refer to (Melinscak and Bach, 2020) as an example of how to use Bayesian Design Optimisation to plan experiments to decide between data-generating models, rather than parameters of the same model in different participant groups, as done here.

 2. The results also hinge on the (somewhat related but not equivalent) assumption that the data-generating process is exactly the same as the model used to analyse the data (i.e. the measurement model). The appropriateness of the measurement model can be assessed wihtout knowing the data-generating process, by running an experiment with a manipulation the effect of which is (somewhat) known, and testing different data analysis methods in terms of how well they reproduce the known effect. This method is known as experiment-based calibration (Bach et al., 2023, 2020), and the relevant metric as retrodictive validity (Bach and Melinscak, 2020).

 3. The notation of many integrals is incorrect; sometimes the integration variable („dtheta“) is not stated (e.g. eq. 1-2), often the integration interval is missing or incorrectly replaced with the integration variable (e.g. eq. 1 should read int_{integration_interval} rather than int_{integration_variable}.).

 4. It is rather confusing that the same symbol P is used to refer to multiple functions depending on what argument they take. That is, P(theta) is supposed to be something different from, let’s say, P(omega). This is an abuse of mathematical notation. A common and useful convention in mathematical statistics is to differentiate probability distributions by subscript (e.g. P_X|Y, P_theta, P_omega, etc.). Psychological statistics literature is obviously rife with this abuse of notation, but in a sophisticated and heavily mathematical manuscript like this, the authors can clearly do better.

Is the rationale for developing the new method (or application) clearly explained?

No

Is the description of the method technically sound?

Partly

Are the conclusions about the method and its performance adequately supported by the findings presented in the article?

Partly

If any results are presented, are all the source data underlying the results available to ensure full reproducibility?

Yes

Are sufficient details provided to allow replication of the method development and its use by others?

Partly

Reviewer Expertise:

NA

I confirm that I have read this submission and believe that I have an appropriate level of expertise to confirm that it is of an acceptable scientific standard, however I have significant reservations, as outlined above.

## References

[1] : Psychophysiological modelling and the measurement of fear conditioning. https://doi.org/10.1016/j.brat.2020.103576 10.1016/j.brat.2020.103576 PMC707875032087391

[2] : Consensus design of a calibration experiment for human fear conditioning. https://doi.org/10.1016/j.neubiorev.2023.105146 10.1016/j.neubiorev.2023.105146 PMC1061840736990370

[3] Melinscak, F., Bach, D.R., 2020. Computational optimization of associative learning experiments. PLoS Comput Biol. https://doi.org/10.1371/journal.pcbi.1007593.10.1371/journal.pcbi.1007593PMC696491531905214

# Response

**Published:** 2026-04-25

**Authors:** Sarah Schreiber

**Affiliations:** University of Oxford, Oxford, England, UK

*Schreiber et al. give a tutorial-style introduction into, and worked example of, the established framework of Bayes Factor Design Analysis, applied in the context of a multi-armed bandit task. No new method is developed. Thus, the original scientific contribution appears relatively small, but the worked example provides a welcome illustration of this (suite of) method(s) for students of the field. My major concern is that two assumptions of the method are not sufficiently discussed; there are also a couple of minor technical concerns.*  

**Response**: We are thankful to Reviewer 2 for a generally positive evaluation of our work.

*Point 1 & 2:*

*Any results hinge on the assumption of a specific data-generating process. If the true data-generating process (in a future experiment) is not the same as the one assumed in the simulation, then results such as required group size etc. will be misleading. The authors might want to refer to (Melinscak and Bach, 2020) as an example of how to use Bayesian Design Optimisation to plan experiments to decide between data-generating models, rather than parameters of the same model in different participant groups, as done here.*

*The results also hinge on the (somewhat related but not equivalent) assumption that the data-generating process is exactly the same as the model used to analyse the data (i.e. the measurement model). The appropriateness of the measurement model can be assessed wihtout knowing the data-generating process, by running an experiment with a manipulation the effect of which is (somewhat) known, and testing different data analysis methods in terms of how well they reproduce the known effect. This method is known as experiment-based calibration (Bach et al., 2023, 2020), and the relevant metric as retrodictive validity (Bach and Melinscak, 2020).*  

**Response**: We thank the reviewer for highlighting this important point. In response, we have expanded the limitations section to more clearly discuss the dependence of BFDA on the assumed generative model. We now also explicitly refer to Bayesian Design Optimisation as an approach for comparing competing data-generating models, and to experiment-based calibration for evaluating the validity of the measurement model.

**Revised Section:** “The dependence on an accurate generative model is a general limitation of using BFDA, as design recommendations are conditional on that model. If the true data-generating process deviates from the specified model, conclusions about the optimal design may be inaccurate. In settings with multiple competing computational models aiming to describe a process, Bayesian Design Optimisation can be used to compare candidate data-generating models (Melinscak and Bach, 2020). Independently, the adequacy of the analysis model can be evaluated empirically using experiment-based calibration (Bach & Melinscak, 2020; Bach et al., 2020; Bach et al., 2023).”  

*Point 3.*

*The notation of many integrals is incorrect; sometimes the integration variable („dtheta“) is not stated (e.g. eq. 1-2), often the integration interval is missing or incorrectly replaced with the integration variable (e.g. eq. 1 should read int_{integration_interval} rather than int_{integration_variable}.).*

**Response**: We thank the reviewer for pointing this out and have revised the equations accordingly.  

*Point 4.*

*It is rather confusing that the same symbol P is used to refer to multiple functions depending on what argument they take. That is, P(theta) is supposed to be something different from, let’s say, P(omega). This is an abuse of mathematical notation. A common and useful convention in mathematical statistics is to differentiate probability distributions by subscript (e.g. P_X|Y, P_theta, P_omega, etc.). Psychological statistics literature is obviously rife with this abuse of notation, but in a sophisticated and heavily mathematical manuscript like this, the authors can clearly do better.*  

**Response**: We thank the reviewer for this helpful comment. We have revised the notation throughout the manuscript to avoid overloading the symbol  and making clear when probability distributions are being used.    

**References** 1. Dominik R. Bach, Filip Melinscak: Psychophysiological modelling and the measurement of fear conditioning. | Publisher Full Text 2. Dominik R. Bach ,Juliana Sporrer,Rany Abend,Tom Beckers,Joseph E. Dunsmoor,Miquel A. Fullana,Matthias Gamer,Dylan G. Gee,Alfons Hamm,Catherine A. Hartley,Ryan J. Herringa,Tanja Jovanovic,Raffael Kalisch,David C. Knight,Shmuel Lissek,Tina B. Lonsdorf,Christian J. Merz, Mohammed Milad, Jayne Morriss, Elizabeth A. Phelps, Daniela Schiller: Consensus design of a calibration experiment for human fear conditioning. | Publisher Full Text Melinscak, F., Bach, D.R., 2020. Computational optimization of associative learning experiments. PLoS Comput Biol. https://doi.org/10.1371/journal.pcbi.1007593.

# Reviewer report: Reviewer response for version 1

**DOI:** 10.21956/wellcomeopenres.24565.r104233

**Published:** 2024-10-21

**Authors:** Brandon S Coventry

**Affiliations:** 1 University of Wisconsin-Madison, Madison, Wisconsin, USA

Dear authors,

 It was a pleasure to read your article detailing Bayes Factor design of experiments and its incorporation into latent variable modeling. Much well deserved interest has recently been received to incorporate Bayesian methods to the design of experiments, and I’m pleased to see your method adding to the conversation. I believe that modeling experiments during the experimental design phase with well-defined models of decision processes can certainly help bound and inform the BFDA process in meaningful ways. However, there are several areas in the manuscript that require further refinement to enhance both its readability and the broader adoption of your model and methodology.

 I have outlined my feedback below, categorizing my suggestions into general, article-wide comments, as well as specific line-by-line revisions tied to particular sections of the text.

**General Comments**

 I believe that the description of the MAB task needs much better description. As it stands, it’s placed in the context of a pain model that is never described, and the definition of a painful stimulus is vague at best.

 I also believe that this article often verges into vague descriptions of what is being done. I’ve tried to highlight sections below, but I believe portions can be rewritten to explicitly state what is being done. This will greatly increase readability.

 It should be noted that the use of Bayes factors in model comparisons can be tricky, with the potential to reduce meaningful Bayesian inference to a modified p-value. Many argue that the power of Bayesian thinking lies in using prior knowledge to build the best possible models and to avoid the temptation to just compare every model available. For a good discussion of these caveats, see (Gelman and Rubin, 1995; Gelman and Shalizi, 2013). Providing a discussion of and context to BFDA may help allay some of these criticisms of BF analyses.

 I also believe this article would benefit greatly from following the Bayesian analysis reporting guidelines (BARG) for article clarity and reproducibility (Kruschke, 2021).

**Specific Comments**

**Abstract**

 Interest in the use of Bayesian statistics I believe extends well beyond just the fact that one can incorporate prior knowledge. While that is certainly a powerful component of Bayesian design, other advantageous features include the fact that inference is performed using the data on hand without implicit assumptions on population distributions, the ability to easily handle data distributions which are not strictly normal, direct quantification of uncertainty under inference, and the ability to iteratively update hypotheses as data is observed.

**Introduction**

 “Designing an effective experiment can be challenging due to the number of parameters that need to be considered.” While this is true, I believe this is a bit reductive, potentially to the point of trivializing design of experiments (DOE). Rewording of text and adding appropriate discussion of the goals of DOE would add important context to the reader, especially those relatively new to the science and admittedly art of DOE.

 “To improve a study’s effective-ness, it is recommended to conduct a thorough prospective

 design analysis rather than relying purely on a retrospective approach”.

 While true, I believe it’s important to mention preregistration as part of the current initiatives to improve reproducibility. For a thorough discussion of the pros and cons of preregistration, see (Vize et al., 2024).

 “Bayesian methods are gaining recognition for their advantages, as they allow the incorporation of prior knowledge into statistical processes.”

 Again, I think much more can be said about the advantages of Bayesian approaches. See comments above in abstract section, and the following references (Blackwell and Ramamoorthi, 1982; Gelman and Shalizi, 2013; Kruschke, 2013, 2010).

 “Traditionally, sample size determination methods rely on frequentist statistics, but there has been an ongoing critique among statisticians and methodologists regarding these approaches.”

 While true, I’d argue that the primary goal of DOE is not just to determine sample size, but to determine sample size to ensure an experiment has the statistical power to detect if a desired effect is truly present. Sample size is a means to this end of the question experimenters are truly interested in.

 “a-priori”

 Please change to *a priori*

 “BFDA assumes a population with predetermined attributes and conducts simulations on repeated sampling from this population”

 In the context of a tutorial article, I believe concrete examples are going to be needed to best orient the reader to the goals of BFDA. One way to do this is to provide a quick hypothetical experiment, outlining what example predetermined attributes may be, and how one might conduct simulations. Are there experiments where simulations are generally widely available and accepted? What about cases of a novel experiment, how might one deal with model selection? These are questions that should be addressed to best help the reader adopt this framework.

 “However, in psychology and neuroscience, we often deal with latent variables that first need to be inferred from the data collected.”

 Absolutely, and I’m glad this is mentioned. It should be noted that in neuroscience and social sciences, initial passes where latent variables are truly hidden is often addressed with hierarchical Bayesian models. See (Cronin et al., 2010; Gelman, 2006; Gelman and Hill, 2006).

**Methods**

 “The prior distribution P(θ) represents our knowledge about the distribution of θ ahead of data acquisition. The chosen prior can heavily influence the outcome of an analysis and should therefore be chosen carefully, as it will bias the estimation.”

 True, especially if the observed data is weak, in which case priors dominate. However, some guidance of proper prior choice should be detailed in this article. Potentially adding a prior predictive checks to at least one model to help inform the reader of exactly this condition.

 “Once the likelihoods have been calculated for each group, we can compare θ between two groups, for example between patients and healthy controls.”

 Need to clarify for the reader whether you mean a point estimate of θ or the distribution of θ.

 “Figure 1 panel A”

 I think this figure has the promise of being very good, but is a bit confusing to read as it stands. As an example, the box “Compute the posterior” is positioned right next to, what I believe is the incorporation of the prior into the likelihood to create the posterior, but it appears based on where the box is positioned that you mean to say P(theta) is the posterior.

 “HDPI is a method of obtaining a credible interval for skewed probability distributions.”

 Need to describe credible regions, and I believe you need to redo the definition of the HPDI, as it’s not just for skewed distributions. I think I understand what you mean by skewed here, but as it stands its ambiguous given that one can easily define HPDIs for non-skewed distributions.

 “Figure 1”

 Consider reorganizing the panels of Figure 1 as they are referenced out of order in text, making the figure a little harder to read.

 “The probability of receiving a certain number of painful stimuli in one game is dependent on the choices of the participant on one hand and the reward probabilities of each arm on the other hand.”

 So this is verging into confusion. I understand that MAB problems are used in models of pain as mentioned above, but if this terminology is going to be adapted in the method (painful stimuli), then this model needs to be fleshed out. Otherwise, I don’t not have a well defined view of what MAB model parameters, such as reward and painful stimuli mean. I’d suggest either removing the pain model from the MAB context and explicitly defining model rewards and failures or totally rope the description of the MAB problem into the pain model context.

 “The learning rate α was fixed at 0.1”

 This seems a bit high of a learning rate value. Why was this chosen and if there is appropriate literature, cite it here.

 “Therefore, there are seven possible outcomes, as the agent can receive between zero and six painful stimuli.”

 Again, this needs much better defining.

 “For each choice, there are four possible combinations of arm chosen and aversive or neutral reward. For six free choice trials there are a total of 46 possible paths, or combinations of choices and outcomes.”

 Moving this earlier in the text will help with understanding of the MAB model.

**Results**

 “The use of the BFDA as a method for prospective design analysis allows us to consider a number of factors. However, it is essential to evaluate the algorithm estimating the latent variable as a preliminary step.”

 This needs to be contextualized for understanding. What factors are you considering? What latent variables are you hoping to describe? What specific algorithm do you mean here? This sentence is quite vague. Being quite specific in what results you are showing will help readability greatly.

 “Once the algorithm has been validated, it can be used for the BFDA, in which repeated sampling from a population is simulated and the planned analysis carried out on the sample.”

 A descriptive flow chart figure would be extremely useful here.

 “The individual exploration parameter for each simulated participant was drawn from a normal distribution with population mean μτ”.

 Point to the exact parameter referenced in the methods, as right now this is vague.

 “The overall fit of the regression model was statistically significant (F(3,496) = 136300, p < 0.001,R2 = 0.999)”

 This regression model isn’t described in the methods. Please detail what exactly is done here. It’s also unclear why a frequentist approach here was used to validate the algorithm, given that the aim was a Bayes factor approach. This needs better detailing for why this was done.

 “250 simulations were run for each combination of Δμτ ranging from 0 to 1 in increments of 0.05 and sample size per group ranging from 10 to 60 in increments of 2.”

 Please make this a little clearer. What simulation? Markov decision process? Is this MAB RL?

 Better descriptions of what is being done need to be added.

 “Figure 2”

 This is interestingly. What is happening at 0.05? It seems that BF dominate predictions except in this small window. When there’s 0 difference in the mean, Bayes is doing create, but minimal and not moderate uncertainty proves problematic?

 “For Δμτ ≠ 0 this probability is the probability of wrongfully accepting the null hypothesis, which corresponds to the type II error.”

 In Bayesian models, type I/II errors are generally not considered for a variety of reasons related to how Bayesian inference is performed. Bayesian approaches instead talk of Type M or Type S errors generally. You can derive a type I/II error for Bayesian approaches, but care must be taken in doing so. See (Gelman and Carlin, 2014).

 ”Figure 3”

 Please label color map axis in this figure.

 “This is supported by positive correlations between P(BF10 > 10) with n and ngames (rs(23) = 0.74, p < 0.001;rs(7) = 0.62, p < 0.001) and negative correlations between P(BF10 < 1/10) with n and ngames (rs(23) = –0.49, p < 0.001, rs(7) = –0.31, p < 0.001”

 I think a stronger rational needs to be given here for mixing Bayesian and frequentist approaches to understanding this problem. These are fundamentally different statistical paradigms with inference performed differently. While they may come to similar conclusions, care needs to be taken here.

**Discussion**

 “n this work, we used BFDA to analyse the effect of sample size, number of games per participant, and effect size on the probability of obtaining significant evidence to support a null or alternative hypothesis,and the probabilities of incorrectly supporting either hypoth-esis **.”**

 This needs to be contextualized for experiments that are modeled by MAB problems, and not strictly true for all possible experimental designs. I think this is where the real power of this model lies. If one is running experiments with similar designs and can, using prior knowledge, place reasonable predictions around model parameters, then one has a really nice and cheap way of assessing bounds and providing reasonable sample size prediction. However, the moment the experimental design is not MAB-like, then these results may not be strictly true.

 “examine latent variables in relation to BFDA.”

 Again, specific examples of this from results would be extremely beneficial.

 “In the example we used an uninformative prior”

 This needs to be placed in methods section to orient the reader to what priors are being used. Add in uniform prior parameters as well. See (Kruschke, 2021).

**Works Cited**

 Blackwell, D.L., Ramamoorthi, R.V., 1982. A Bayes but Not Classically Sufficient Statistic. The Annals of Statistics 10, 1025–1026.

 Cronin, B., Stevenson, I.H., Sur, M., Körding, K.P., 2010. Hierarchical Bayesian Modeling and Markov Chain Monte Carlo Sampling for Tuning-Curve Analysis. Journal of Neurophysiology 103, 591–602. https://doi.org/10.1152/jn.00379.2009

 Gelman, A., 2006. Prior distributions for variance parameters in hierarchical models (comment on article by Browne and Draper). Bayesian Analysis 1. https://doi.org/10.1214/06-BA117A

 Gelman, A., Carlin, J., 2014. Beyond Power Calculations: Assessing Type S (Sign) and Type M (Magnitude) Errors. Perspect Psychol Sci 9, 641–651. https://doi.org/10.1177/1745691614551642

 Gelman, A., Hill, J., 2006. Data Analysis Using Regression and Multilevel/Hierarchical Models, 1st ed. Cambridge University Press, Cambridge, United Kingdom.

 Gelman, A., Rubin, D.B., 1995. Avoiding Model Selection in Bayesian Social Research. Sociological Methodology 25, 165. https://doi.org/10.2307/271064

 Gelman, A., Shalizi, C.R., 2013. Philosophy and the practice of Bayesian statistics: *Philosophy and the practice of Bayesian statistics*. Br J Math Stat Psychol 66, 8–38. https://doi.org/10.1111/j.2044-8317.2011.02037.x

 Kruschke, J.K., 2021. Bayesian Analysis Reporting Guidelines. Nat Hum Behav 5, 1282–1291. https://doi.org/10.1038/s41562-021-01177-7

 Kruschke, J.K., 2013. Bayesian estimation supersedes the t test. Journal of Experimental Psychology: General 142, 573–603. https://doi.org/10.1037/a0029146

 Kruschke, J.K., 2010. What to believe: Bayesian methods for data analysis. Trends in Cognitive Sciences 14, 293–300. https://doi.org/10.1016/j.tics.2010.05.001

 Vize, C.E., Phillips, N.L., Miller, J.D., Lynam, D.R., 2024. On the Use and Misuses of Preregistration: A Reply to Klonsky (2024). Assessment 10731911241275256. https://doi.org/10.1177/10731911241275256

Is the rationale for developing the new method (or application) clearly explained?

Partly

Is the description of the method technically sound?

Partly

Are the conclusions about the method and its performance adequately supported by the findings presented in the article?

Partly

If any results are presented, are all the source data underlying the results available to ensure full reproducibility?

Yes

Are sufficient details provided to allow replication of the method development and its use by others?

No

Reviewer Expertise:

Bayesian Statistics, Neuroscience, Neural Engineering, Reinforcement Learning in Neuroscience

I confirm that I have read this submission and believe that I have an appropriate level of expertise to confirm that it is of an acceptable scientific standard, however I have significant reservations, as outlined above.

## References

[1] : Hierarchical Bayesian modeling and Markov chain Monte Carlo sampling for tuning-curve analysis. *J Neurophysiol* .2010;103(1) : 10.1152/jn.00379.2009 591-602 10.1152/jn.00379.2009 19889855 PMC2807240

[2] : Prior distributions for variance parameters in hierarchical models (comment on article by Browne and Draper). *Bayesian Analysis* .2006;1(3) : 10.1214/06-BA117A 10.1214/06-BA117A

[3] : Beyond Power Calculations: Assessing Type S (Sign) and Type M (Magnitude) Errors. *Perspect Psychol Sci* .2014;9(6) : 10.1177/1745691614551642 641-51 10.1177/1745691614551642 26186114

[4] : Avoiding Model Selection in Bayesian Social Research. *Sociological Methodology* .1995;25: 10.2307/271064 10.2307/271064

[5] : Philosophy and the practice of Bayesian statistics. *Br J Math Stat Psychol* .2013;66(1) : 10.1111/j.2044-8317.2011.02037.x 8-38 10.1111/j.2044-8317.2011.02037.x 22364575 PMC4476974

[6] : Bayesian Analysis Reporting Guidelines. *Nat Hum Behav* .2021;5(10) : 10.1038/s41562-021-01177-7 1282-1291 10.1038/s41562-021-01177-7 34400814 PMC8526359

[7] : Bayesian estimation supersedes the t test. *J Exp Psychol Gen* .2013;142(2) : 10.1037/a0029146 573-603 10.1037/a0029146 22774788

[8] : What to believe: Bayesian methods for data analysis. *Trends Cogn Sci* .2010;14(7) : 10.1016/j.tics.2010.05.001 293-300 10.1016/j.tics.2010.05.001 20542462

[9] : On the Use and Misuses of Preregistration: A Reply to Klonsky (2024). *Assessment* .2024; 10.1177/10731911241275256 10731911241275256 10.1177/10731911241275256 39258834 PMC11871985

[10] Gelman, A., Hill, J., 2006. Data Analysis Using Regression and Multilevel/Hierarchical Models, 1st ed. Cambridge University Press, Cambridge, United Kingdom.

[11] Blackwell, D.L., Ramamoorthi, R.V., 1982. A Bayes but Not Classically Sufficient Statistic. The Annals of Statistics 10, 1025–1026.

# Response

**Published:** 2026-04-25

**Authors:** Sarah Schreiber

**Affiliations:** University of Oxford, Oxford, England, UK

*It was a pleasure to read your article detailing Bayes Factor design of experiments and its incorporation into latent variable modeling. Much well deserved interest has recently been received to incorporate Bayesian methods to the design of experiments, and I’m pleased to see your method adding to the conversation. I believe that modeling experiments during the experimental design phase with well-defined models of decision processes can certainly help bound and inform the BFDA process in meaningful ways. However, there are several areas in the manuscript that require further refinement to enhance both its readability and the broader adoption of your model and methodology.*

**Response**: We are thankful to Reviewer 1 for a generally positive evaluation of our work.

*Point 1.*

*I believe that the description of the MAB task needs much better description. As it stands, it’s placed in the context of a pain model that is never described, and the definition of a painful stimulus is vague at best.* *I also believe that this article often verges into vague descriptions of what is being done. I’ve tried to highlight sections below, but I believe portions can be rewritten to explicitly state what is being done. This will greatly increase readability.*

**Response:** We thank the reviewer for this feedback. We have revised the description of the multi-armed bandit (MAB) task to improve clarity and specificity. The revised text now provides a clearer definition of the task structure, the exploration-exploitation trade-off, and the nature of the task outcomes. We also explicitly describe the pain-related application of the binary MAB, specifying the delivery versus absence of a painful stimulus (e.g., electrocutaneous stimulation), and we clarify the fixed task horizon and trial structure.

**Revised Sections:** “To validate the analysis pipelines and to provide a concrete example for the proposed approach, a binary two-armed bandit task was simulated. The MAB is a widely-used paradigm to investigate the exploration-exploitation dilemma in behavioural science (Danwitz *et al.*, 2022; Daw *et al.*, 2006; Gershman, 2019), in which the agent (for example the participant or a reinforcement learning agent) repeatedly chooses between multiple actions or “arms”. Over time the agent learns the reward probabilities associated with each arm. To maximise the cumulative outcome across trials, the agent must balance exploring the available arms in order to reduce uncertainty about their outcomes (exploration) and using the information they have gathered so far to choose the optimal option (exploitation).” “The outcomes can be continuous, for example in the form of a scalar monetary reward, where participants learn the reward distribution (e.g. mean and uncertainty), or binary, such as the presence versus absence of a food reward or a negative (aversive) stimulus. The binary version of the MAB task is often used in pain research, where a painful stimulus (e.g., an electrocutaneous shock) is administered. The two possible outcomes would be the delivery of an aversive (e.g. painful) stimulus and its absence (Krypotos *et al.*, 2022a; Krypotos *et al.*, 2022b). In the computational modelling of these outcomes, each outcome must be assigned a numerical value. Here, the outcome of each choice was coded as 0 or -1, where -1 represents an aversive outcome.” ***“*** To facilitate differentiation between exploration and exploitation, the agent has information on both options available to them prior to their first choice, so that the agent possesses information to base their exploitation on (Wilson et al., 2014). This is achieved by presenting the agent with four actions and their immediate results, after which they are free to make their own choices. The task was implemented with a fixed horizon of 10 choices, defining the number of trials within each game, in that each game consisted of four observed trials followed by 6 free choice trials. The outcome probabilities were combinations of the probabilities 0.1, 0.3, and 0.9, with the probability assigned to each arm changing after each game.”

*Point 2. It should be noted that the use of Bayes factors in model comparisons can be tricky, with the potential to reduce meaningful Bayesian inference to a modified p-value. Many argue that the power of Bayesian thinking lies in using prior knowledge to build the best possible models and to avoid the temptation to just compare every model available. For a good discussion of these caveats, see (Gelman and Rubin, 1995; Gelman and Shalizi, 2013). Providing a discussion of and context to BFDA may help allay some of these criticisms of BF analyses.*  

**Response**: We thank the reviewer for this comment. We have added a discussion that contextualises our use of Bayes factors within the broader Bayesian literature, explicitly acknowledging common concerns about threshold-based interpretations. We clarify that Bayes factors are used here as a pragmatic tool for design analysis, rather than as a replacement for full Bayesian inference.

**Revised Section:** “It is important to highlight that while this analysis summarizes Bayes factors using common thresholds, their interpretation is not inherently discrete. The primary strength of Bayes factors lies in their continuous nature and in their role within a broader Bayesian framework that emphasizes principled model construction and the use of prior knowledge (Aczel et al., 2020; Coventry & Bartlett, 2024; Gelman & Rubin, 1995; Gelman & Shalizi, 2013). However, in the present work, Bayes factors are used specifically as a pragmatic tool for design analysis, where threshold-based summaries aid decision-making. In retrospective analyses, Bayes factors should still be interpreted within a full Bayesian framework rather than being reduced to a modified p-value.”  

*Point 3.*

*I also believe this article would benefit greatly from following the Bayesian analysis reporting guidelines (BARG) for article clarity and reproducibility (Kruschke, 2021).*  

**Response** *: * We thank the reviewer for this helpful suggestion. We have now incorporated several relevant elements of the Bayesian Analysis Reporting Guidelines for reporting retrospective Bayesian inference to improve the clarity and transparency of our approach for prospective Bayes factor design analysis. Specifically, we now more clearly state the goals of the analysis, explicitly define the observed data and inferred parameters, and clarify the specification and justification of the priors used. We believe these additions improve readability and reproducibility while remaining appropriate to the prospective focus of the present work.

**Revised Sections:** **“**The goal of this work is a prospective design analysis, specifically to evaluate how sample size, number of games per participant, and effect size influence Bayes factors and parameter recovery for a latent exploration parameter in a multi-armed bandit task. We first analysed the accuracy of Bayesian parameter estimations of latent variables, within a population, as well as between two groups, using simulated behavioural data in a multi-armed bandit task (Krypotos *et al.*, 2024). In a second step, we combined BFDA with simulations of behavioural data to explore the relationship between sample size and the strength of evidence for both null and alternative hypothesis.” **“**To summarise, the primary outcome per game is the number of aversive outcomes (0–6), while the main inferred parameter is the exploration parameter τ. The between group comparison focusses on the difference in the exploration parameter Δμ_τ. For both tau and the difference in population means (Δμ_τ), we used a discrete uniform prior defined over a bounded parameter space (τ ∈ [0.01, 3] and Δμ_τ ∈ [-3, 3]).”

**Specific Comments**

Abstract

*Point 4.*

*Interest in the use of Bayesian statistics I believe extends well beyond just the fact that one can incorporate prior knowledge. While that is certainly a powerful component of Bayesian design, other advantageous features include the fact that inference is performed using the data on hand without implicit assumptions on population distributions, the ability to easily handle data distributions which are not strictly normal, direct quantification of uncertainty under inference, and the ability to iteratively update hypotheses as data is observed.*

**Response**: We thank the reviewer for this comment. Due to abstract word limits, these points could not be fully included. However, we have reworded the Abstract to better reflect the broader advantages of the Bayesian framework and expanded the Introduction to highlight several of the reviewer’s suggestions.

**Revised Section:** Abstract: “Bayesian statistics offers a flexible framework that supports iterative updating of hypotheses and the incorporation of prior information, amongst other advantages.” Introduction: “Concurrently, Bayesian methods are gaining recognition for their advantages, which include the incorporation of prior knowledge into statistical processes, the ability to quantify evidence for both null and alternative hypotheses, accommodate non-normal data, and directly represent uncertainty through probability distributions (Jeffreys, 1935; Jeffreys, 1961; Wagenmakers, 2007; Blackwell & Ramamoorthi, 1982;Gelman & Shalizi, 2013; Kruschke, 2010; Kruschke, 2013).”

Introduction:

*Point 5.*

 “ *Designing an effective experiment can be challenging due to the number of parameters that need to be considered.” While this is true, I believe this is a bit reductive, potentially to the point of trivializing design of experiments (DOE). Rewording of text and adding appropriate discussion of the goals of DOE would add important context to the reader, especially those relatively new to the science and admittedly art of DOE*.

**Response**: We thank the reviewer for this comment and have revised the Introduction accordingly.

**Revised Section:** “Designing an effective experiment is a multifaceted process that begins with formulating clear research questions and hypotheses, selecting appropriate methodologies, and aligning design choices with the underlying theoretical framework. Beyond these conceptual considerations, the appropriate analyses and practical parameters must be carefully determined to ensure valid, reliable, and adequately powered statistical inference (Czitrom 2012; Garud et al., 2017; Tůmová et al., 2018).  

*Point 6.*

*“To improve a study’s effectiveness, it is recommended to conduct a thorough prospective design analysis rather than relying purely on a retrospective approach”. While true, I believe it’s important to mention preregistration as part of the current initiatives to improve reproducibility. For a thorough discussion of the pros and cons of preregistration, see (Vize et al., 2024).*  

**Response**:  We thank the reviewer for this suggestion. We have revised the manuscript to explicitly reference preregistration as part of broader reproducibility initiatives.

**Revised Section: ** “Prospective design analyses can help optimize the use of available resources, which has become increasingly important considering recent concerns about 'research waste' (Ioannidis *et al.*, 2014; Macleod *et al.*, 2014; Storz-Pfennig, 2017). These approaches align with efforts to improve reproducibility and replicability, such as preregistration and sharing of code and data (Munafò et al., 2017; Vize et al., 2025). As BFDA requires the primary analysis to be specified a priori, it integrates well with these practices.”  

*Point 7.*

*“Bayesian methods are gaining recognition for their advantages, as they allow the incorporation of prior knowledge into statistical processes.” Again, I think much more can be said about the advantages of Bayesian approaches. See comments above in abstract section, and the following references (Blackwell and Ramamoorthi, 1982; Gelman and Shalizi, 2013; Kruschke, 2013, 2010).*  

**Response**: We agree with the reviewer and have revised this section to more clearly emphasize the general advantages of Bayesian statistics and their role in Bayes Factor Design Analysis.

**Revised Sections:** “Concurrently, Bayesian methods are gaining recognition for their advantages, which include the incorporation of prior knowledge into statistical processes, the ability to quantify evidence for both null and alternative hypotheses, accommodate non-normal data, and directly represent uncertainty through probability distributions (Jeffreys, 1935; Jeffreys, 1961; Wagenmakers, 2007; Blackwell & Ramamoorthi, 1982;Gelman & Shalizi, 2013; Kruschke, 2010; Kruschke, 2013).  

*Point 8.*

*“Traditionally, sample size determination methods rely on frequentist statistics, but there has been an ongoing critique among statisticians and methodologists regarding these approaches.” While true, I’d argue that the primary goal of DOE is not just to determine sample size, but to determine sample size to ensure an experiment has the statistical power to detect if a desired effect is truly present. Sample size is a means to this end of the question experimenters are truly interested in.*  

**Response**: We thank the reviewer for this important clarification. We have revised the Introduction to more clearly emphasize that sample size determination is a means to ensuring adequate statistical power to detect meaningful effects, rather than an end in itself.  

**Revised Section: ** “Designing an effective experiment is a multifaceted process that begins with formulating clear research questions and hypotheses, selecting appropriate methodologies, and aligning design choices with the underlying theoretical framework. Beyond these conceptual considerations, the appropriate analyses and practical parameters must be carefully determined to ensure valid, reliable, and adequately powered statistical inference (Czitrom 2012; Garud et al., 2017; Tůmová et al., 2018). **”**   “Traditionally, the focus of predictive analyses has been on determining the sample size required to ensure adequate statistical power to detect meaningful effects, which is a useful step to ensure the quality and validity of the experiment and all conclusions drawn from it. Methodologies for determining sample sizes have long primarily relied on frequentist statistics, but there has been an ongoing critique among statisticians and methodologists regarding these approaches. Among the main concerns are the common misinterpretation of *p*-values and significance testing (Benjamin *et al.*, 2018; Gigerenzer, 2004; Morrison & Henkel, 2017; Wagenmakers, 2007; Wagenmakers *et al.*, 2018).”

*Point 9.*

*“a-priori”. Please change to a priori*  

**Response**: This has been revised.

**Revised Section: ** “BFDA evaluates the distribution of the Bayes factors for a given experimental design, providing a powerful alternative to frequentist a priori power analyses.”

*Point 10.*

*“BFDA assumes a population with predetermined attributes and conducts simulations on repeated sampling from this population”. In the context of a tutorial article, I believe concrete examples are going to be needed to best orient the reader to the goals of BFDA. One way to do this is to provide a quick hypothetical experiment, outlining what example predetermined attributes may be, and how one might conduct simulations. Are there experiments where simulations are generally widely available and accepted? What about cases of a novel experiment, how might one deal with model selection? These are questions that should be addressed to best help the reader adopt this framework.*  

**Response**: We appreciate the reviewer’s suggestion to include a hypothetical example. However, as the manuscript develops the example of the MAB in detail throughout the remainder of the tutorial, we believe that introducing an additional hypothetical scenario would be beyond the scope of the current article and may create confusion for the reader. Instead, we have revised this section to highlight the goal of BFDA, to clarify the notion of population-level assumptions and elaborated on how distributional properties may be specified in both established and novel paradigms.  

**Revised Section:** “BFDA assumes that the variable of interest is defined at the population level, and that observations are sampled from this population. In well-established paradigms, distributional properties and effect size estimates of the variable at population level can be informed by previous research. In novel paradigms, standard distributions (e.g. the normal distribution) may be assumed and sensitivity analyses across possible effect sizes can be used to assess robustness (Schönbrodt & Wagenmakers, 2018). For each sample generated under this population model, the comparative evidence between the null hypothesis and the alternative hypothesis is measured by calculating the Bayes factor. Repeating this process yields a distribution of Bayes factors, which can be used to evaluate and compare design approaches and thus optimise the experimental setup.”

*Point 11.*

*“However, in psychology and neuroscience, we often deal with latent variables that first need to be inferred from the data collected.”Absolutely, and I’m glad this is mentioned. It should be noted that in neuroscience and social sciences, initial passes where latent variables are truly hidden is often addressed with hierarchical Bayesian models. See (Cronin et al., 2010; Gelman, 2006; Gelman and Hill, 2006).*  

**Response**: We thank the reviewer for calling these references to our attention and have revised the section accordingly.

**Revised Section: ** “However, in psychology and neuroscience, we often deal with latent variables that first need to be inferred from the data collected. Such cases are commonly addressed using computational models (e.g. of behaviour or neural responses), including hierarchical Bayesian models, which allow latent parameters to be estimated while accounting for variability at multiple levels (Cronin et al., 2010; Gelman, 2006; Gelman & Hill, 2006). **”** Methods *.*

*Point 12.*

*“The prior distribution P(θ) represents our knowledge about the distribution of θ ahead of data acquisition. The chosen prior can heavily influence the outcome of an analysis and should therefore be chosen carefully, as it will bias the estimation.” True, especially if the observed data is weak, in which case priors dominate. However, some guidance of proper prior choice should be detailed in this article. Potentially adding a prior predictive checks to at least one model to help inform the reader of exactly this condition.*  

**Response**: We are grateful for this comment. While an extensive treatment and comparison of prior specification is beyond the scope of this tutorial, we have added references to relevant literature to provide readers with further guidance.

**Revised Section: ** “The chosen prior can heavily influence the outcome of an analysis and should therefore be chosen carefully, as it will bias the estimation. When prior knowledge about the underlying distribution is limited, researchers often employ uninformative or weakly informative priors, which can nevertheless reflect plausible bonds for the parameters. Given the subjective nature of priors, their selection should be transparently reported and sufficiently motivated. In addition, sensitivity analyses can be conducted to evaluate the robustness of results with respect to the chosen prior. For further discussions on priors, refer to further literature such as Gelman (2006), Van Dongen (2006), or Stefan (2019).”

*Point 13.*

*“Once the likelihoods have been calculated for each group, we can compare θ between two groups, for example between patients and healthy controls.”*

*Need to clarify for the reader whether you mean a point estimate of θ or the distribution of θ.*

**Response**: We thank the reviewer for this comment and have clarified that the comparison refers to distributions rather than point estimates.

**Revised Section: ** “Once the likelihoods have been calculated for each group, we can compare the distribution of *θ* between two groups, for example between patients and healthy controls.”

*Point 14.*

*“Figure 1 panel A”*

*I think this figure has the promise of being very good, but is a bit confusing to read as it stands. As an example, the box “Compute the posterior” is positioned right next to, what I believe is the incorporation of the prior into the likelihood to create the posterior, but it appears based on where the box is positioned that you mean to say P(theta) is the posterior.*  

**Response**: We appreciate these observations regarding Figure 1. This figure has now been revised to improve clarity and understanding for the reader. The figure legend has also been revised.  

*Point 15.*

*“HDPI is a method of obtaining a credible interval for skewed probability distributions.”*

*Need to describe credible regions, and I believe you need to redo the definition of the HPDI, as it’s not just for skewed distributions. I think I understand what you mean by skewed here, but as it stands its ambiguous given that one can easily define HPDIs for non-skewed distributions.*  

**Response**: We thank the reviewer for this helpful clarification. We have revised the definition of the HPDI to more accurately describe it and its average length.

**Revised Section: ** “A further analysis evaluates the accuracy of the estimation of the difference between two groups by assessing the estimation error and the average length of the highest probability density interval (HPDI) to aid in determining an optimal sample size. The HPDI is a type of credible interval, representing a range of values within which the parameter has a certain probability of falling (e.g. 95%). Among all possible intervals, the HPDI is the credible interval that includes the highest probability densities. Therefore, a 95% HDPI would include the range of values with the highest densities and 95% probability. The interval is defined as  with  An example of a HPDI is shown in Figure 1d. The length of the HPDI can serve as a measurement of uncertainty, with narrower intervals reflecting more precise estimates. The average length of the posterior’s *HPDI* can be calculated in a two-step approach ( Joseph & Bélisle, 1997). Let *l'* be the length of the *HPD*_1–α_ interval for a given dataset *Y _i_*. The average length l* can be calculated from *l'* by multiplying it by the probability of *Y _i_* being the outcome for this measurement and integrating over all possible outcomes *γ* = [ *Y*_1_, *Y*_2_,...] .  is the posterior predictive density and can be computed as .”

*Point 16.*

*“Figure 1”*

*Consider reorganizing the panels of Figure 1 as they are referenced out of order in text, making the figure a little harder to read.*

**Response**: This figure has now been revised to improve clarity and understanding for the reader. In-text references to Figure 1 have been revised to ensure that all panels are referred to in order.  

*Point 17.*

*“The probability of receiving a certain number of painful stimuli in one game is dependent on the choices of the participant on one hand and the reward probabilities of each arm on the other hand.”*

*So this is verging into confusion. I understand that MAB problems are used in models of pain as mentioned above, but if this terminology is going to be adapted in the method (painful stimuli), then this model needs to be fleshed out. Otherwise, I don’t not have a well-defined view of what MAB model parameters, such as reward and painful stimuli mean. I’d suggest either removing the pain model from the MAB context and explicitly defining model rewards and failures or totally rope the description of the MAB problem into the pain model context*.  

**Response**: We recognize that combining the MAB framework with a pain-related example may have been unclear. The aversive stimulus was chosen due to its relevance in the pain literature. To improve clarity, we revised the explanation and changed the terminology to consistently distinguish the general MAB model from its experimental instantiation, replacing “painful stimulus” with “aversive outcome” in the modelling context. This is also addressed in the response to point 1.

**Revised Sections:** “The outcomes can be continuous, for example in the form of a scalar monetary reward, where participants learn the reward distribution (e.g. mean and uncertainty), or binary, such as the presence versus absence of a food reward or a negative (aversive) stimulus. The binary version of the MAB task is often used in pain research, where a painful stimulus (e.g., an electrocutaneous shock) is administered. The two possible outcomes would be the delivery of an aversive (e.g. painful) stimulus and its absence (Krypotos *et al.*, 2022a; Krypotos *et al.*, 2022b). In the computational modelling of these outcomes, each outcome must be assigned a numerical value. Here, the outcome of each choice was coded as 0 or -1, where -1 represents an aversive outcome.” “The binary two-armed bandit task was simulated as described and the number of draws with an aversive outcome was recorded. The probability of receiving a certain number of aversive outcomes in one game is dependent on the choices of the participant on one hand and the reward probabilities of each arm on the other hand.”

*Point 18.*

*“The learning rate α was fixed at 0.1”*

*This seems a bit high of a learning rate value. Why was this chosen and if there is appropriate literature, cite it here.*  

**Response**: We thank the reviewer for this comment. The value α = 0.1 was selected as a fixed illustrative parameter to facilitate the simulations. We have adjusted the wording to make the considerations clearer.

**Revised Section: ** “In the present simulations, the learning rate α was arbitrarily fixed at 0.1 for illustrative purposes. Although learning rates may vary, particularly in aversive contexts (Krypotos et al., 2022b; Wang et al., 2018), our conclusions are not contingent on this specific choice.”

*Point 19.*

*“Therefore, there are seven possible outcomes, as the agent can receive between zero and six painful stimuli.” Again, this needs much better defining.* *“For each choice, there are four possible combinations of arm chosen and aversive or neutral reward. For six free choice trials there are a total of 46 possible paths, or combinations of choices and outcomes.” Moving this earlier in the text will help with understanding of the MAB model.*  

**Response**: We thank the reviewer for these helpful comments. We have revised and restructured the entire MAB section, moving the description of possible choice-outcome combinations and clarifying the wording to improve overall comprehensibility.

Results

*Point 20.*

*“The use of the BFDA as a method for prospective design analysis allows us to consider a number of factors. However, it is essential to evaluate the algorithm estimating the latent variable as a preliminary step.” This needs to be contextualized for understanding. What factors are you considering? What latent variables are you hoping to describe? What specific algorithm do you mean here? This sentence is quite vague. Being quite specific in what results you are showing will help readability greatly*.  

**Response**: We revised this section for clarity, more clearly specifying the goals of the BFDA, the outcome parameters, and the analytical approach.

**Revised Section: ** “The use of the BFDA as a method for prospective design analysis allows us to systematically evaluate design parameters, including sample size, number of games, task-specific features such as number of arms and aversive outcome probabilities, and the planned analysis approach. In our example experiment of the binary MAB with aversive outcomes, our hypothetical question is to compare the exploration parameter τ between two groups. This is a latent parameter which influences the agents’ choices (eq.14). Our analysis consists of three main steps summarized in Figure 1c. First, we verify the estimations of population mean for one population. Next, we compare the population means between two group by examining the distribution of Bayes factors, which indicate whether there is evidence for or against a difference in population mean between groups. This analysis is carried out to determine possible effect sizes and the effect of experimental parameters, such as the number of participants, as well as the number of games per participant on the evidence. In the last step, we analyse potential errors when estimating a difference in means between groups and how they are affected by the experimental parameters (number of participants and number of games per participant).”

*Point 21.*

*“Once the algorithm has been validated, it can be used for the BFDA, in which repeated sampling from a population is simulated and the planned analysis carried out on the sample.”*

*A descriptive flow chart figure would be extremely useful here.*  

**Response**: We thank the reviewer for this helpful suggestion. Figure 1c presents a flowchart of the validation and BFDA procedures. We have now adjusted the text to improve clarity.

**Revised Section: ** “In our example experiment of the binary MAB with aversive outcomes, our hypothetical question is to compare the exploration parameter τ between two groups. This is a latent parameter which influences the agents’ choices (eq.14). Our analysis consists of three main steps summarized in Figure 1c. First, we verify the estimations of population mean for one population. Next, we compare the population means between two group by examining the distribution of Bayes factors, which indicate whether there is evidence for or against a difference in population mean between groups.”

*Point 22.*

*“The individual exploration parameter for each simulated participant was drawn from a normal distribution with population mean μτ”. Point to the exact parameter referenced in the methods, as right now this is vague.*  

**Response**: We have adjusted the text to refer to the softmax algorithm, where τ was first introduced and to include more information on the population.

**Revised Section:** **“**For each simulated participant, the individual exploration parameter τ, as defined in the softmax algorithm (14), was drawn from a normal distribution with population mean *μ _τ_* ranging from 0.05 to 1 in increments of 0.05, and a standard deviation of *σ* = 0.02. The goal was to then estimate the population mean within the Bayesian framework as described (see Figure 1c, Validation of estimations of population mean).”

*Point 23.*

*“The overall fit of the regression model was statistically significant (F(3,496) = 136300, p < 0.001,R2 = 0.999)” This regression model isn’t described in the methods. Please detail what exactly is done here. It’s also unclear why a frequentist approach here was used to validate the algorithm, given that the aim was a Bayes factor approach. This needs better detailing for why this was done.*

**Response**: We thank the reviewer for this important point. We have revised the Methods section to explicitly describe the regression model used and clarify its role. We now state that the regression analysis is a descriptive check assessing the correspondence between the algorithm’s estimates and the known generative values in simulation. The analysis is not part of the Bayesian inferential framework and is not intended to validate the Bayes factor approach. The frequentist formulation was used solely for transparency and ease of interpretation in this auxiliary verification step.

**Revised Section:** “To evaluate the accuracy of this estimation, we compared the point estimates calculated as stated in (5) with the true exploration parameters. This was done using a linear regression model with point estimates as the dependent variable and true mean difference, sample size, and their interaction as predictors. The regression model, as well as the subsequent models, were estimated using ordinary least squares in the seaborn python package (Seabold & Perktold, 2010). These regression analyses and correlations are descriptive summaries of the observed relationships and are not part of the Bayesian inferential framework or the Bayes factor-based design analysis. They are used solely as a descriptive check to assess whether the simulated data contain informative signal regarding the accuracy of the estimated population means. We then calculated the mean squared estimation error (MSEE) and evaluated its relationship with sample size using Spearman’s rank-order correlation. We also computed a regression model with the MSEE as the dependent variable and the sample size, true population mean, and their interaction as predictors, allowing for nonlinear relationships. After inspection, the sample size was logarithmically transformed, and the population mean was exponentially transformed. The scaling constants used in these transformations were determined in prior curve-fitting procedures.” “Next, we analysed the estimated difference in mean between the two populations by comparing the estimated difference between the two populations to the true difference Δ *μ*_τ_. 250 simulations were run for each combination of Δ *μ*_τ_ ranging from 0 to 1 in increments of 0.05 and sample size per group ranging from 10 to 58 in increments of 2. A linear regression model with point estimates of the difference as the dependent variable and true mean difference, sample size, and their interaction as predictors was used as a descriptive summary of the correspondence between estimated and generative values. Again, these analyses are not part of Bayesian inferential framework. The MSEE was calculated for the estimated difference and its relationship with sample size and mean difference was examined using a regression model with MSEE as the dependent variable and the sample size, true mean difference, and their interaction as predictors, incorporating nonlinear transformations of the predictors. Log transformation was applied to the sample size, and exponential transformation was applied to the difference in population mean, with scaling constants established in earlier curve-fitting operations.”

*Point 24.*

*“250 simulations were run for each combination of Δμτ ranging from 0 to 1 in increments of 0.05 and sample size per group ranging from 10 to 60 in increments of 2.”*

*Please make this a little clearer. What simulation? Markov decision process? Is this MAB RL?*

*Better descriptions of what is being done need to be added.*  

**Response**: We thank the reviewer for this comment. We have expanded the description of the simulation procedure to clarify that each replication involved simulating participant choices within MAB reinforcement learning framework, followed by computation of the Bayes factor as part of the BFDA. The revised text now more explicitly outlines the sequential steps of the simulation and analysis process.

**Revised Section:** **“**To evaluate the effects of sample size and difference in means on the evidence for a difference between groups, we performed the BFDA. First, we drew random samples of the exploration parameter τ from the two populations with different means, with each sampled value representing one simulated participant. Behavioural outcomes within the binary MAB were then generated based on τ, and the distribution  was computed as described in Equation 6, from which the Bayes factor was calculated (Equation 8). This procedure was repeated 250 times for each combination of the sample size of each group ranging from 10 to 58 in increments of 2 and difference in means ranging from 0 to 0.95 in increments of 0.05. To determine the population means for a given mean difference, the mean of the first population was randomly sampled from a uniform distribution between 0 and , and the mean of the second population was defined by adding the specified difference. The Bayes factor was calculated for each of the 250 simulations, yielding a distribution of Bayes factors reflecting the evidence for or against a difference in means at each combination of sample size and mean difference.”

*Point 25.*

*“Figure 2”*

*This is interestingly. What is happening at 0.05? It seems that BF dominate predictions except in this small window. When there’s 0 difference in the mean, Bayes is doing create, but minimal and not moderate uncertainty proves problematic?*  

**Response**: We thank the reviewer for this observation. Because the null hypothesis is specified as a point hypothesis (i.e., Δμτ = 0), even extremely small deviations from zero imply that the null is technically false. In this region of very small effect sizes, the Bayes factor may not strongly favour the alternative unless the sample size is sufficiently large. As a result, moderate evidence can appear unstable in this narrow range, reflecting the difficulty of distinguishing very small effects from zero. We have explicitly added this fact to the text.

**Revised Section:** “With an increase of Δ *μ*_τ_ and *n* the Bayes factors are more likely to support the correct hypothesis and the stronger the evidence in favour of the correct hypothesis. When the difference in means is zero, the evidence correctly supports the null hypothesis. However, even very small deviations from zero technically contradict the null hypothesis, which can challenge the algorithm. Detecting and strongly supporting such minimal differences requires very large sample sizes. **”**

*Point 26.*

*“For Δμτ ≠ 0 this probability is the probability of wrongfully accepting the null hypothesis, which corresponds to the type II error.”*

*In Bayesian models, type I/II errors are generally not considered for a variety of reasons related to how Bayesian inference is performed. Bayesian approaches instead talk of Type M or Type S errors generally. You can derive a type I/II error for Bayesian approaches, but care must be taken in doing so. See (Gelman and Carlin, 2014).*  

**Response**: We thank the reviewer for this comment and have revised the relevant sections to explicitly define and label Type S and Type M errors.

**Revised Sections:** “Similarly, we can calculate the probability of receiving a Bayes factor *BF*_10_ smaller than 1/3 which would indicate at least moderate evidence in favour of the null hypothesis (Figure 3b). When this occurs despite a true nonzero mean difference, it constitutes a Type S (sign) error in the Bayesian framework.”   “To gain an understanding of how the Bayes factor relates to the true estimate of the difference in mean, we can determine the magnitude error for those simulations that result in a Bayes factor greater than 10. The magnitude error (Type M error) is calculated by dividing the difference between the estimated value and the true difference in mean by the true difference in mean. This reflects an overestimation of the true effect size in statistically significant results (Gelman & Carlin, 2014).”

*Point 27.*

*”Figure 3”*

*Please label color map axis in this figure.*

**Response**: The colour map axis has been labelled in Figure 3 as requested. This change has also been applied to Figure 4.

*Point 28.*

*“This is supported by positive correlations between P(BF10 > 10) with n and ngames (rs(23) = 0.74, p < 0.001;rs(7) = 0.62, p < 0.001) and negative correlations between P(BF10 < 1/10) with n and ngames (rs(23) = –0.49, p < 0.001, rs(7) = –0.31, p < 0.001”*

*I think a stronger rational needs to be given here for mixing Bayesian and frequentist approaches to understanding this problem. These are fundamentally different statistical paradigms with inference performed differently. While they may come to similar conclusions, care needs to be taken here.*

**Response**: We thank the reviewer for raising this important point. As in Point 23, the reported correlations are not intended as inferential statistics but as descriptive summaries of simulation outcomes. We have clarified this distinction in the manuscript to explicitly separate these summaries from the Bayesian inferential framework.  

**Revised Section:** “These regression analyses and correlations are descriptive summaries of the observed relationships and are not part of the Bayesian inferential framework or the Bayes factor-based design analysis. They are used solely as a descriptive check to assess whether the simulated data contain informative signal regarding the accuracy of the estimated population means.”  

Discussion

*Point 29.*

*“In this work, we used BFDA to analyse the effect of sample size, number of games per participant, and effect size on the probability of obtaining significant evidence to support a null or alternative hypothesis, and the probabilities of incorrectly supporting either hypothesis **.”***

*This needs to be contextualized for experiments that are modeled by MAB problems, and not strictly true for all possible experimental designs. I think this is where the real power of this model lies. If one is running experiments with similar designs and can, using prior knowledge, place reasonable predictions around model parameters, then one has a really nice and cheap way of assessing bounds and providing reasonable sample size prediction. However, the moment the experimental design is not MAB-like, then these results may not be strictly true.*

**Response**: We thank the reviewer for this insightful comment. We agree that the present findings are specific to experiments modelled within a multi-armed bandit framework. We have revised the manuscript to more clearly contextualise the results within this task structure and to avoid overgeneralization.

**Revised Section:** “In this work, we used BFDA in the example of a binary multi-armed bandit (MAB) task with aversive outcomes to analyse the effect of sample size, number of games per participant, and effect size on the probability of obtaining significant evidence to support a null or alternative hypothesis, and the probabilities of incorrectly supporting either hypothesis.”   “It is important to note that these results are specific to the task used. Although some findings may generalize to similar MAB learning tasks, they do not necessarily extend to other experimental paradigms.”

*Point 30.*

*“examine latent variables in relation to BFDA.”*

*Again, specific examples of this from results would be extremely beneficial.*  

**Response**: We thank the reviewer for this comment. We have revised the text accordingly to improve clarity.

**Revised Sections:** “The example of a multi-armed bandit (MAB) task with prior information was used to examine latent variables in relation to BFDA. Specifically, we considered a hypothetical experiment designed to assess differences in the exploration parameter (τ), estimated via the softmax decision-rule, between two groups. We showed how sample size, number of games per participant and effect size influence the probability of obtaining evidence for group differences in τ.”   “We found that it can be challenging to determine the difference in means between two groups. Large sample sizes are needed for a strong probability of detecting evidence in favour of difference, where present. If there is strong evidence supporting the alternative hypothesis, the probability of overestimating the difference in means is relatively high. In the example we used an uninformative prior, but depending on the experiment and existing literature, this should be adjusted. A well-informed prior can lead to higher probabilities of detecting a difference between groups.”

*Point 31.*

*“In the example we used an uninformative prior”*

*This needs to be placed in methods section to orient the reader to what priors are being used. Add in uniform prior parameters as well. See (Kruschke, 2021).*

**Response**: We thank the reviewer for pointing this out. We have revised the methods section to explicitly state the prior specification, including the parameters of the uniform prior.

**Revised Section:** **“**For both tau and the difference in population means (Δμ_τ), we used a discrete uniform prior defined over a bounded parameter space (τ ∈ [0.01, 3] and Δμ_τ ∈ [-3, 3]).”

**References** 1. Cronin B, Stevenson IH, Sur M, et al.: Hierarchical Bayesian modeling and Markov chain Monte Carlo sampling for tuning-curve analysis. *J Neurophysiol. *2010; **103** (1): 591-602 PubMed Abstract | Publisher Full Text 2. Gelman A: Prior distributions for variance parameters in hierarchical models (comment on article by Browne and Draper). *Bayesian Analysis. *2006; **1** (3): | Publisher Full Text 3. Gelman A, Carlin J: Beyond Power Calculations: Assessing Type S (Sign) and Type M (Magnitude) Errors. *Perspect Psychol Sci. *2014; **9** (6): 641-51 PubMed Abstract | Publisher Full Text 4. Gelman A, Rubin D: Avoiding Model Selection in Bayesian Social Research. *Sociological Methodology. *1995; **25** | Publisher Full Text 5. Gelman A, Shalizi CR: Philosophy and the practice of Bayesian statistics. *Br J Math Stat Psychol. *2013; **66** (1): 8-38 PubMed Abstract | Publisher Full Text 6. Kruschke JK: Bayesian Analysis Reporting Guidelines. *Nat Hum Behav. *2021; **5** (10): 1282-1291 PubMed Abstract | Publisher Full Text 7. Kruschke JK: Bayesian estimation supersedes the t test. *J Exp Psychol Gen. *2013; **142** (2): 573-603 PubMed Abstract | Publisher Full Text 8. Kruschke JK: What to believe: Bayesian methods for data analysis. *Trends Cogn Sci. *2010; **14** (7): 293-300 PubMed Abstract | Publisher Full Text 9. Vize CE, Phillips NL, Miller JD, et al.: On the Use and Misuses of Preregistration: A Reply to Klonsky (2024). *Assessment. *2024; 10731911241275256 PubMed Abstract | Publisher Full Text Gelman, A., Hill, J., 2006. Data Analysis Using Regression and Multilevel/Hierarchical Models, 1st ed. Cambridge University Press, Cambridge, United Kingdom. Blackwell, D.L., Ramamoorthi, R.V., 1982. A Bayes but Not Classically Sufficient Statistic. The Annals of Statistics 10, 1025–1026.
